# Simulating Polar Bear Energetics during a Seasonal Fast Using a Mechanistic Model

**DOI:** 10.1371/journal.pone.0072863

**Published:** 2013-09-03

**Authors:** Paul D. Mathewson, Warren P. Porter

**Affiliations:** 1 Nelson Institute for Environmental Studies, University of Wisconsin-Madison, Madison, Wisconsin, United States of America; 2 Department of Zoology, University of Wisconsin-Madison, Madison, Wisconsin, United States of America; University of Alberta, Canada

## Abstract

In this study we tested the ability of a mechanistic model (Niche Mapper™) to accurately model adult, non-denning polar bear (*Ursus maritimus*) energetics while fasting during the ice-free season in the western Hudson Bay. The model uses a steady state heat balance approach, which calculates the metabolic rate that will allow an animal to maintain its core temperature in its particular microclimate conditions. Predicted weight loss for a 120 day fast typical of the 1990s was comparable to empirical studies of the population, and the model was able to reach a heat balance at the target metabolic rate for the entire fast, supporting use of the model to explore the impacts of climate change on polar bears. Niche Mapper predicted that all but the poorest condition bears would survive a 120 day fast under current climate conditions. When the fast extended to 180 days, Niche Mapper predicted mortality of up to 18% for males. Our results illustrate how environmental conditions, variation in animal properties, and thermoregulation processes may impact survival during extended fasts because polar bears were predicted to require additional energetic expenditure for thermoregulation during a 180 day fast. A uniform 3°C temperature increase reduced male mortality during a 180 day fast from 18% to 15%. Niche Mapper explicitly links an animal’s energetics to environmental conditions and thus can be a valuable tool to help inform predictions of climate-related population changes. Since Niche Mapper is a generic model, it can make energetic predictions for other species threatened by climate change.

## Introduction

Ecological effects of recent climate warming on plants and animals have been documented for nearly every major taxonomic group around the globe from the equator to the poles [1]. The greatest temperature changes are occurring in the Arctic, resulting in declining sea ice concentrations [2], [3], upon which many Arctic species depend for various aspects of their life history. The polar bear (*Ursus maritimus*) is among the most ice dependent Arctic marine mammals [4], and is listed as vulnerable by the IUCN [5] and threatened under the United States Endangered Species Act [6] because of sea ice habitat loss. The ability to predict impacts of climate change is critical for successful proactive management of the species.

There has been an accelerating declining trend in Arctic sea ice extent in all months since 1979, a trend that is expected to continue in the future, and which has already impacted polar bear habitat quality [3], [7], [8]. Declines in sea ice extent and other alterations to the polar bear’s sea ice habitat are predicted to have a multitude of effects on the bears [9]–[11]. Population, body size and birth rate declines, presumably as a result of sea ice changes, have already been documented in some polar bear populations [9], [12], [13].

Polar bears use the sea ice as a platform for hunting seals, their primary prey. However, across a portion of their range the sea ice melts completely in the summer, forcing polar bears ashore for months at a time [14]. During these ice-free periods, polar bears in these areas do not have access to seals and must rely on stored energy reserves for survival [14], [15]. Therefore, declining sea ice affects polar bears in these areas primarily through nutritional stress resulting from a combination of less time available to hunt prey and accumulate body energy reserves, and longer periods of time relying on body reserves for energy [12], [16]. The sea ice is breaking up progressively earlier in the summer, forcing bears in these populations ashore for longer periods until the autumn freeze-up [17]–[19]. This also leaves less time to feed in the early summer, which is the most important time of year for depositing the fat reserves necessary to survive extended periods of fasting [13].

Managing and protecting species affected by climate change depends on the ability to accurately predict potential effects of a changing environment [20], [21], and bioenergetic mechanistic models can provide a means of linking climate to biogeography [21], [22]. Commonly used correlative approaches (i.e., associating current species occurrence with environmental variables) are limited with respect to predicting responses to climate change because extrapolations to novel environments may be prone to error [21], [23], [24]. Bioenergetic models do not rely on current distribution information, but rather model an animal’s interaction with its surrounding microclimate environment to predict energetic constraints on distribution. This approach allows animals to be modeled in novel environments without risking erroneous extrapolation. These models thus represent a complementary method to help evaluate and strengthen correlative predictions when assessing the potential impacts of global warming [21].

Recent studies have provided predictions about polar bear survival under increasingly long fasts [25], [26]. However, these studies do not consider potential differences of polar bear interactions with the environment and what this may mean for energetic expenditure in different fasting scenarios. Here, we test the ability of a mechanistic model that directly links animal energetics to microclimate conditions (Niche Mapper™; [27]–[33]) to model non-denning polar bear energetics in a seasonal fast during the ice-free period. We use the western Hudson Bay population as a model because it is well-studied and is already experiencing adverse impacts from climate change, offering a preview of what other populations may later experience [12].

To test the model, we incorporated a polar bear body composition model developed by Molnár et al. [34] to predict weight loss for non-denning polar bears based on Niche Mapper’s metabolic requirement predictions for a 120 day fast (average fast length in the 1980’s and 1990’s when prior studies measured weight loss in this population). The model’s predictions are compared to empirical data of weight loss from field studies of the population. We then use the model to predict polar bear survival and energetic expenditure during a180 day fast (simulated future fast) to investigate potential impacts of climate change on this population.

## Methods

### Niche Mapper Overview

Niche Mapper is a mechanistic model that has been previously described in detail and tested with a wide range of animal species including birds and mammals ranging from mice (*Mus* spp.) to oryx (*Oryx leucoryx*) through comparisons of observed to predicted metabolic rates and observed distributions across the landscape to predicted distributions based on energetic requirements (e.g., [27]–[33]). This will be the first application of Niche Mapper to a bear species. Briefly, Niche Mapper consists of two sub-models: a microclimate model that calculates environmental conditions at animal height and a biophysical/behavioral model. The biophysical model uses hourly environmental information from the microclimate model and user-supplied physiological, morphological, and behavioral characteristics of a given animal to estimate hourly energetic demands for an animal at steady state with its surroundings.

The microclimate model calculates the air, temperature, wind speed, relative humidity, solar radiation, and ground temperature that the animal experiences [35]. The model fits a sinusoidal curve to user-specified maximum and minimum daily air temperatures, wind speeds, cloud cover, and relative humidity to estimate hourly values. Default minimum temperature and wind speed and maximum relative humidity and cloud cover are assumed to occur at sunrise. Maximum temperature and wind speed and minimum relative humidity and cloud cover are assumed to occur one hour after solar noon. Clear sky solar radiation is calculated based on date, hour of day, and geographic location, and is then adjusted to account for cloud cover and overhead vegetation.

Long wavelength thermal radiation from the clear sky and clouds are computed separately using empirical air temperature correlations from the literature [36], [37]. Thermal radiation from the ground is computed from substrate (bare ground/ground vegetation/snow/ice) hourly temperatures derived from the numerical solution of the one dimensional finite difference transient heat balance equation for the ground.

The biophysical model then calculates radiative (Q_rad_), convective (Q_conv_), solar (Q_sol_), and evaporative (respiratory: Q_resp_ and cutaneous: Q_evap_) heat fluxes between the animal and its microenvironment to solve a heat balance equation (Eq. 1) to estimate a metabolic rate, Q_gen,_ that allows the animal to maintain its core temperature:
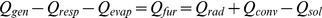
(1)


That is, metabolic heat production (Q_gen_), less evaporative heat loss from the respiratory system (Q_resp_) and the skin (Q_evap_), must equal heat flow through the fur by parallel conduction and radiation processes (Q_fur_) and the net heat flux with the outside environment (Q_rad_+Q_conv_ − Q_sol_) for the animal to be in thermal steady state with its environment. Any deviation from the equality will result in a net heat loss or heat gain, rendering the animal unable to maintain its body temperature.

Individual body parts (head, neck, torso, legs) are modeled as geometric shapes (e.g., cylinders, spheres, ellipsoids) with known heat transfer properties and temperature profile equations used to solve the heat balance equation (see, e.g., [38], [39]) Each body part is modeled as having up to three concentric layers: 1) a solid central geometry of heat generating tissue; 2) a surrounding layer of insulating subcutaneous fat, modeled as a hollow geometry; 3) a surrounding layer of insulating fur, modeled as a hollow geometry. Metabolic heat produced by the central tissue layer must be transferred through the fat layer to the skin surface, where is it either dissipated via cutaneous evaporation or transferred through the fur layer. Heat then travels though the fur and is lost to the environment via radiation or convection, while at the same time heat from solar radiation can be absorbed in this layer.

Thus, the total heat flux through the animal to the environment is a function of the temperature gradients set up from the core out to the environment (core to skin; skin to fur-air interface; fur-air interface to environment). The skin temperature and fur-air interface temperatures, which are unknown for an animal maintaining a certain core temperature in certain environmental temperatures, are solved for simultaneously using the known temperature profile equations for the geometric shape being modeled and information about the animal’s core temperature, the dimensions and thermal properties of each layer, and the external temporally varying microclimate conditions (See Supporting Information for details on the heat balance solution procedure).

For every hour of each model day, the heat balance is solved for each body part and then summed to provide the total metabolic rate for the entire animal that will allow it to maintain its core temperature in that hour’s environmental conditions. From this summed metabolic rate, a respiratory heat loss is calculated based on the amount of oxygen needed to fuel the animal’s metabolic rate and the animal’s oxygen extraction efficiency. Users can apply multiples of basal metabolic rate to simulate activity, specifying what percentage of the activity energy is considered in the heat balance calculations (i.e., 0% means that the activity is 100% efficient with no excess heat produced; 99% means that 99% of the additional metabolic rate is lost as heat and needs to be considered in the heat balance).

If the summed metabolic rate falls outside user-specified variation from a target metabolic rate (i.e., basal metabolic rate×activity multiplier) in the hour being modeled, thermoregulatory options are engaged to prevent overheating or, if the animal is cold, to reduce metabolic expenditure on heat production. Behavioral options are engaged first. The user can allow the animal to seek shade or enter a burrow to seek cooler environmental conditions if the animal is too hot (i.e., the summed metabolic rate is below the target level) or make postural changes such as curling up so the legs are tucked into the torso to minimize surface area for heat exchange with the environment if the animal is too cold (c.f. [40]).

If behavioral thermoregulation is insufficient or unavailable, physiological options are engaged in the following order: increased or decreased flesh thermal conductivity (simulating vasodilation or vasoconstriction of peripheral blood vessels), increased or decreased core temperature (simulating temporary and bounded heat storage or body cooling), increased amount of surface area that is wet (simulating sweating), and decreased oxygen extraction efficiency (to simulate panting).

The heat balance is re-solved after each incremental thermoregulatory change until a balance is reached for the target metabolic rate or thermoregulatory options are exhausted, resulting in the metabolic rate closest to the target rate that satisfies the heat balance for that hour. Hourly metabolic rates are then summed to calculate a daily metabolic rate.

### Microclimate Model Parameterization

We used climate information from Churchill, Manitoba, Canada to model the microclimate conditions experienced by polar bears in the western Hudson Bay population during the ice-free period. Daily maximum and minimum temperatures were obtained from Canada’s National Climate Data and Information Archive (“CNCDIA”; [41]). We used the average daily maximum and minimum temperatures from 1977–2006 for each day modeled. When modeling the future extended fast, we modeled two scenarios: keeping the 1977–2006 average temperatures and adding 3°C to the average daily maximum and minimum temperatures from 1977–2006 to simulate a generic warmer climate. This increase was chosen as a reasonable predicted temperature increase in the region [42], [43].

Daily maximum and minimum wind speeds and relative humidities were unavailable from the Data Archive. Within a given month, the average monthly values from 1971–2000 were used as both the maximum and minimum wind speeds and relative humidities. Daily cloud cover was set to range from 30–100% as estimated from CNCDIA Climate Normals 1971–2000 for Churchill, where 82% of hours between June and December had >30% cloud cover [41]. The ground was assumed to be clear of snow from June through September, and covered in snow from October through December, based on CNCDIA Climate Normals 1971–2000 for Churchill [41]. Sensitivity analyses for various microclimate model inputs are presented in supplementary material (Figs. S1, S2; Table S1).

### Biophysical Model Parameterization: Overall Body Composition

We used a polar bear body composition model from Molnár et al. [34] to initialize model bears for our fasting simulations. The body composition model divides the bear’s total mass into structural mass and storage mass. Structural mass was approximated using the bear’s straight-line body length (Eq. 16 in [34]), and could not be catabolized for energy. Storage mass, the remainder of the animal’s total mass, was available to be used for energy [34]. Storage mass was subdivided into fat mass and lean body mass [34]. Energy stores available to bears of different sizes were estimated for adult males and females using equations 18E and 18C, respectively, from Molnár et al. [34].

To simulate a heterogeneous population, we modeled adult males with straight-line body lengths of 2.15, 2.3, and 2.45 meters, and adult females with straight-line body lengths of 1.85, 2.0, and 2.1 meters. These lengths are centered on the average adult body lengths for the western Hudson Bay population [44] and are similar to the body sizes used to validate the body composition model in Molnár et al. [34].

For each straight-line body length, we simulated bears starting the fast in a range of body conditions. Total body mass of adult female Hudson Bay polar bears ranged from 114–366% of estimated structural mass, and adult male total body mass ranged from 115–321% [34]. Approximately 95% of bears had total body masses greater than 150% of the estimated structural mass [34]. From this information, we created three classes of bears – “poor”, “average”, and “excellent” – where total body masses were 1.5, 2.25 and 3 times the estimated structural body mass, respectively. Table 1 summarizes the initial body composition and energy stores for polar bears modeled in our fasting simulations.

**Table 1:** Initial body composition of bears used in the fasting simulations.

	Straight-LineBody Length (m)	Total Body Mass (kg)Pr/Avg/Ex	StructuralMass (kg)	Initial Fat Mass(kg) Pr/Avg/Ex	Initial Non-StructuralLean Body Mass (kg)Pr/Avg/Ex	Initial Energy Stores(MJ) Pr/Avg/Ex
**Females**	1.85	142/213/283	95	30/74/119	17/44/71	1236/3091/4945
	2.00	179/269/359	120	37/94/150	22/56/89	1562/3905/6248
	2.10	208/311/415	138	43/108/174	26/64/103	1808/4521/7233
**Males**	2.15	223/334/445	148	33/82/130	42/104/167	1448/3619/5791
	2.30	273/409/545	182	40/100/160	51/127/204	1772/4430/7089
	2.45	330/494/659	220	48/121/193	62/154/246	2142/5355/8569

### Biophysical Model Parameterization: Modeling Individual Body Parts

Niche Mapper breaks animals down into a series of body parts (head, neck, torso, front legs, back legs) represented by simple geometric shapes because each body part will interact differently with the environment due to differences in shape, size, and insulation. The neck, torso, and legs were modeled as cylinders, and the head was modeled as a truncated cone. Body part lengths and horizontal and vertical diameters were measured by ruler on photographs of Western Hudson Bay polar bears [45] and then scaled to actual animal size using straight-line body length (scaling factor = animal straight-line body length/sum of head, neck and torso lengths). For example, if the head, neck and torso lengths measured on the photographs summed up to 11.5 cm the scaling factor used to model a 230 cm long bear would be 20.

For each body part, a subroutine in Niche Mapper subtracted twice the user-input for fur depth from the scaled up diameters to obtain a skin diameter, and calculated geometric volumes of each body part using the skin radii and lengths. The total animal mass was then apportioned to each body part according to its volume (e.g., if the torso accounted for 70% of the total calculated volume of the animal, the torso would be allocated 70% of the animal’s total mass). Fat mass was allocated similarly, except we did not allow any subcutaneous fat to be deposited on the head.

Body part density was then calculated by dividing the body part mass by the calculated volume. If the density deviated from ∼1000 kg/m^3^ (our estimation), adjustments were made to the skin diameters to reach the desired density. This ensured that same length bears in different body conditions would have the same body part lengths, but different body part diameters to reflect mass differences.

Each body part was next divided into a solid central cylinder of heat generating flesh surrounded by a hollow cylinder of subcutaneous fat of uniform thickness. Of the total body fat, 75% was considered to be subcutaneous [46], thus contributing to the insulating fat layer. Subcutaneous fat volume (assuming a fat density of 901 kg/m^3^ [47]) was subtracted from the body part volume to get a volume for the heat generating flesh cylinder. Flesh radii were then calculated using the flesh volume and the body part length, and the thickness of the insulating fat layer was calculated as the difference between the skin radius and flesh radius.

Skin surface areas were calculated for each body part from the skin radii and body part lengths. Fur depth was added to the skin radii for fur surface area calculations. The torso had the end areas for cylinders representing the neck and four legs subtracted from its surface area calculation to account for the joining of these body parts. Similarly the neck and leg surface area calculations do not include end areas due to joining other body parts or being in contact with the ground, nor is the wide bottom end area of the head which is joined to the neck included.

Scaled up dimensions for average sized male and female polar bears as modeled by Niche Mapper are shown in Figure S3. To verify Niche Mapper’s allometry calculations, we compared total surface area and torso circumference for bears as modeled by Niche Mapper to expected surface areas and torso circumferences based on predictive equations derived from measurements on live bears across a wide range of body sizes.

### Biophysical Model Parameterization: Other Inputs and Sensitivity Analyses

Other biophysical model inputs are presented in Table 2, and model sensitivity to these inputs is presented in Fig. S2 and Table S1. Prior to the fasting simulations, we tested Niche Mapper’s calculations against empirical measurements from the literature. First, to test fur temperature calculations we simulated a male polar bear the average size of the two bears on which Best [48] measured radiant fur temperatures while walking on treadmills. Due to the porous nature of fur, radiative exchange occurs simultaneously at various fur depths. Thus, the effective radiant surface temperature is an integration of the temperatures at which radiant energy is exchanged with the environment at various points within the fur layer and is typically higher than the physical fur surface temperature (where convective heat exchange takes place). In our simulations our best estimation was that the fur temperature at 85% of the way along the fur temperature profile from the skin to the fur surface was representative of the effective radiant temperature, which was then used in radiant heat flux calculations. For the simulated bear we targeted a metabolic rate of 2.5× expected basal metabolic rate from the mouse to elephant curve to simulate the treadmill activity and compared Niche Mapper’s calculated torso radiant fur temperature to those reported by Best [48] at different air temperatures and wind speeds.

**Table 2:** Properties used to parameterize the biophysical model.

Parameter	Value Used	Source
Weight	See Table 1	
Core temperature (°C)(Min/Avg/Max)	36/37/39	[48]: Deep body temperatures (35.7∼40.5°C) measured with implanted transmitters on 2 subadult males resting and walking on treadmills.
		[49]: Deep body temperature (37.1∼39.5°C) measured with implanted transmitters on 1 adult female bear resting and walking on a treadmill.
Flesh thermal conductivity(W/mC)	0.4–2.8	[50]: A collection of thermophysical property data on biological media, including a cold living hand (0.34 W/mC) and very warm living skin (2.8 W/mC)., and living skin with blood flow (1.58–7.0 W/mC).
O_2_ extraction efficiency (%)	5–20%	[51]: Mammal O_2_ extraction efficiency is typically around 20%.
		[48]: Visual counts of ventilation rates increased from 10–20 breaths per minute at rest to 105–133 breaths per minute during exercise in one subadult male.
Fur thermal conductivity(W/mC)	0.0628; 7% increasewith each 1 m/swind speed increase	[52]: Measured thermal conductance (mean in calm air: 1.67 W/m^2^C) at various wind speeds on mid-dorsal fur samples from 3 subadult males. No fur depth provided; assuming fur depth was between 30 mm and 50 mm, this would be a calm air thermal conductivity of 0.05 to 0.08 W/mC. Conductance increased linearly (7% for every 1 m/s increase) for wind speeds up to 7 m/s.
		[53]: Thermal conductivity (mean:∼0.05 W/mC) as measured on 6 winter polar bear pelt samples in windless conditions.
		[54]: Thermal conductivity (mean: 0.0628 W/mC) as measured on 3 polar bear pelt dorsal samples in windless conditions.
Hair solar reflectivity	0.61	Own measurements of average reflectivity across 350–2500 nm wavelengths on mid-dorsal fur samples from two polar bear winter pelts from the UW Zoological Museum.
Fur depth (mm) Head/Neck/Torso/Legs	Summer: 10/20/20/20	[55] Measured on two subadult male polar bears in 11 different regions in winter pelt. Head: 3–16 mm; Neck: 64 mm; Torso: 64–133 mm; Legs: 17–37 mm.
	Winter: 15/45/60/35	[53]: Measured on 6 winter polar bear pelt and 4 summer pelt dorsal regions. Winter: 25–55 mm; Summer: 18–30 mm
		[54]: Measured on 3 polar bear pelt dorsal regions: 55–70 mm.
		Own measurements: took top of head (30 mm), neck (44 mm), torso (38 mm) and leg (18–35 mm) measurements on two polar bear fall/winter pelts from the UW Zoological Museum.
		Summer head, neck, and leg measurements are our estimations

We also tested Niche Mapper’s ability to reach a heat balance for a polar bear in simulated denning situation with known size, average metabolic rate, average core temperature and den air temperature [56].

Finally, although we are unaware of any empirical data to compare to, we placed model polar bears in simulated metabolic chambers in which sky, air, and ground temperatures were all set equal to one another, and increased incrementally while other environmental conditions remained constant. This illustrates the impact of ambient temperature on steady state metabolic rates predicted by Niche Mapper using our supplied inputs, as well as the temperature range in which thermoregulatory options allow bears modeled by Niche Mapper to maintain a target basal metabolic rate (Figs. S4, S5).

### Fasting Subroutine

A fasting subroutine was used to simulate weight loss in fasting polar bears. On the first day of the simulated fast, the subroutine estimated the bear’s initial energy stores, as described previously. As a result of microclimate model constraints on the number of input days, fasting periods were divided into four-day intervals, the smallest interval that would allow us to model up to a 180 day fast. The fasting subroutine thus multiplies the energetic demand as calculated using the heat balance described earlier for the day being modeled by the interval length to calculate the total energetic demand for the time period being modeled. This energetic demand is then subtracted from the bear’s total energy reserves, and a new body weight is calculated for use in the next time interval by subtracting the total mass lost to provide energy for the prior interval from total animal mass at the start of the prior time interval. Similarly, the bear’s new body composition is calculated by subtracting the fat and lean body mass used to provide the energy for the prior time interval from the animal’s fat and lean body mass reserves present at the start of the prior time interval. The radial dimensions and fat thickness for each body part are then recalculated to account for the lost fat and lean body mass.

Weight loss for the time interval was calculated assuming that fat reserves accounted for 88.56–94.66% of the bear’s total energetic demands (see below), and that protein in the lean body mass supplies the rest of the energy. We further assumed that the bear’s lean body mass is 22% protein [57], [58]. Once a bear lost all non-structural lean body mass, fat reserves would supply 100% of subsequent energy demands, or vice versa. We kept a running average daily weight loss for the duration of the fast because daily weight loss tended to decrease as the fast progresses due to the reduced metabolic demands of reduced body size. For bears that lost all lean body mass or fat reserves before the end of the fast, the average daily weight loss used in our analyses was taken from the last time interval the bear had both fat and lean body mass reserves in order to compare weight loss to previous field studies. Once energy storage capacity reached 0.0 J, the bear was assumed to have starved to death.

Atkinson and Ramsay [58] found that denning female bears used their fat reserves for 93% of their energetic demands, assuming energy contents of 39.3 MJ/kg for fat and 23.6 MJ/kg for protein. Atkinson et al. [59] report that non-denning bears used fat reserves for an average of 85% of their energetic needs while fasting, assuming the same energy content for fat and protein as Atkinson and Ramsay [58]. Polischuk et al. [60] found that fasting bears lost equal amounts of lean body mass and fat mass.

Molnár et al. [34] used an energy content of 18.0 MJ/kg protein and 39.3 MJ/kg fat when developing their polar bear body composition model. Therefore, we used the same energy content values in our simulations and recalculated the contribution of fat reserves reported in previous studies using 18.0 MJ/kg for protein’s energy content. Assuming that protein accounts for 22% of a polar bear’s lean body mass, fat would contribute 94.66% of the energetic demand in the Atkinson and Ramsay study [58] and 88.56% in the Atkinson et al. study [59]. For bears to lose half their mass in fat and half their weight in lean body mass, as in the Polischuk et al. study [60], fat would supply 90.85% of the bear’s energetic demands. Given these differing results, we simulated fasts using these three different fat reserve contributions to validate the model estimates against field studies: Sim1, Sim2, and Sim3 assume that 94.66%, 90.85%, and 88.56% of energy comes from fat reserves, respectively.

### Fast Simulations

We first simulated a 120 day fast starting on July 29, a typical fast length and onshore date for this population in the 1990s [9], [25], and lasting through November 25. We then increased the fast to 180 days, the potential future fast length used by Molnár et al. [25]. The future fast begins on June 11 and lasts through December 7. We assumed that increased temperatures would cause both an earlier onshore date and a later return to the sea ice. Since sea ice extent has been declining most rapidly during the summer months [61] we added more days to the start of the current fast than we added to the end of it.

The metabolic rate for polar bears resting onshore is unclear. Past studies have reported the polar bear’s ability to reduce metabolic rate as a strategy to survive periods of food shortage [62], [63]. A recent study assigned energetic values to observed lean body and fat losses in fasting polar bears in the Western Hudson Bay to calculate total daily energetic expenditure during the sampling interval [26]. From this a mass-specific equation for daily energetic requirements was derived which was slightly lower than typical mammalian basal metabolic rates [26]. In light of these prior studies, rather than targeting a resting metabolic rate from the mouse-to-elephant curve [64], we used the male energy requirement equation from figure 4b in [26], adjusted to be in J/s rather than kcal/day as our target metabolic rate for all hours of the day. While ashore, adult males and solitary adult females spend more than 90% of the time resting (lying, standing, or moving less than 10m in one direction) [65]. Thus, while the equation from [26] does incorporate some activity, it is likely to be reasonably close to the resting metabolic rate.

Niche Mapper targeted a metabolic rate ±5% of the metabolic rate expected by the equation from [26] for every hour of the day. If the initial metabolic rate calculated in the heat balance was outside this range, thermoregulatory options were engaged, as described above. We did not allow the bears to sweat, and we ran simulations both assuming no shade available (to simulate bears on the coastal plain) and allowing bears to seek shade (to simulate inland bears with access to tree shade).

### Estimating Minimum Energy Density for Survival

We used the model to estimate minimum energy density (total energy stores (MJ) divided by lean body mass (kg); [34]) needed for bears to survive the summer fast in order to compare predicted survivorship to predictions made by Molnár et al. [25]. For both current and projected future environmental conditions, minimum energy density for males and females were estimated by plotting the survival of bears sized from 1.1 times structural mass through the first size that Niche Mapper predicted would survive the fast, incrementally increasing size by 0.1 times structural mass. The resulting best-fit line equation was then used to calculate minimum energy density.

Bears with the same energy density, but with different body lengths have slightly different metabolic requirements per unit body mass, and thus have slightly different survivorship curves. Therefore, we used the average body lengths (230 cm and 200 cm) as proxies for males and females, respectively, when calculating minimum energy density and, in the case of males, comparing predicted survivorship to Molnár et al. [25] predictions.

## Results

### Model Testing

**Figure 1:**
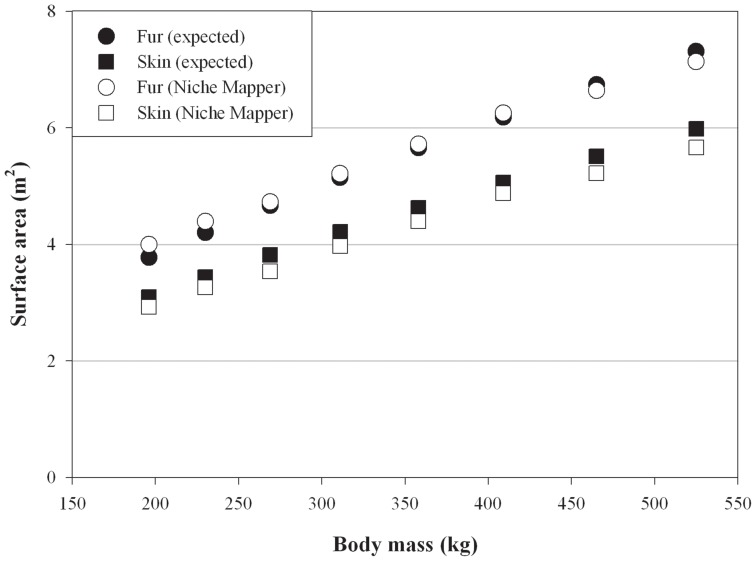
Polar bear surface areas calculated by Niche Mapper compared to expected surface areas. Niche Mapper surface area calculations are for bears modeled in average body condition (total mass = 2.25× structural mass) with straight-line lengths of 1.8–2.5 m (see Text S1 for conversion of body length and condition to total body mass). Best [55] measured surface areas on 18 bears ranging from 11–375 kg and developed predictive equations for skin (surface area = 0.09*mass^0.67^) and fur (surface area = 0.11*mass^0.67^) surface areas (m^2^) as a function of animal mass (kg).

**Figure 2:**
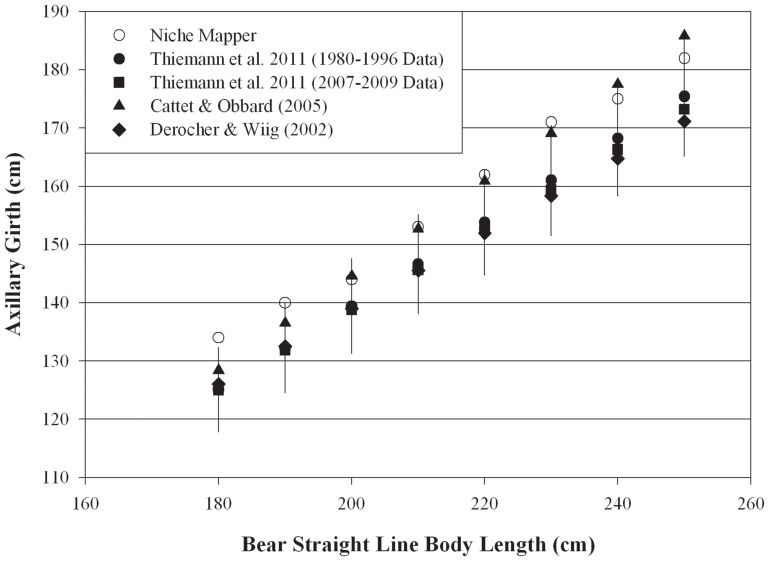
Polar bear torso circumference calculated by Niche Mapper compared to expected axillary girth. Torso cylinder circumferences modeled by Niche Mapper for bears of average body condition (total mass = 2.25× structural mass) compared to expected axillary girth for polar bears of different body lengths based on predictive equations from field study measurements of wild polar bears [66]. The error bars for the Thiemann et al. (1980–1996 data) represent the average 5.84% difference between predicted and actual measurements reported in the study.

**Figure 3:**
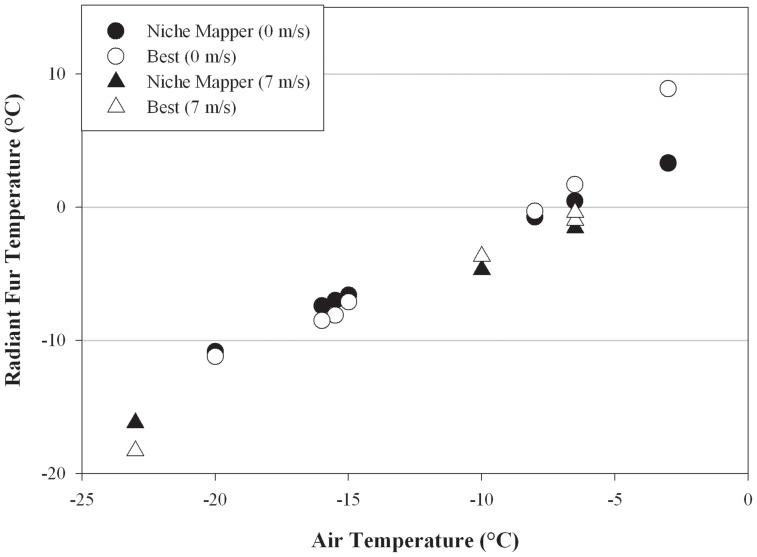
Radiant fur temperature comparison. Comparison of radiant fur temperatures as measured by Best [48] using captive bears and Niche Mapper predictions for a model bears of the size under the same ambient temperatures for no wind and 7 m/s wind speed. To simulate the treadmill activity in the Best study, Niche Mapper targeted a metabolic rate 2.5× expected basal metabolic rate from the mouse to elephant curve. Comparisons were similarly close at 4 m/s wind speed (not shown).

**Figure 4:**
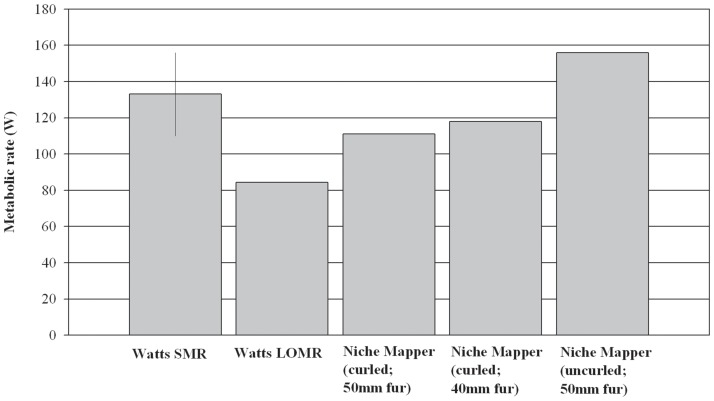
Denning polar bear metabolic rate comparison. Average (± S.E.) standard metabolic rate (SMR) and lowest observed metabolic rate (LOMR) for polar bears under simulated denning conditions [56], compared to Niche Mapper metabolic calculations for simulations of a bear of the same size (204 kg) and core temperature (36.5°C) at steady state with the reported den environmental conditions (−3°C). Niche Mapper attempted to reach the average standard metabolic rate reported in all simulations only by varying flesh thermal conductivity. Bear fur depth was not reported, so the impact of fur depth on Niche Mapper metabolic predictions is shown, as is the impact of curled (legs tucked into torso) and uncurled posture.

Total body surface areas and torso dimensions modeled on bears in average body conditions compare favorably to empirical measurements across a wide range of body sizes (Figs. 1,2). Calculated fur surface areas differed by <6% (average: 1.4%) and skin surface areas by <7% (average: 5.4%) from areas expected based on allometric equations developed from measurements on live bears [55]. Similarly, radiant fur temperatures calculated by Niche Mapper correspond with those measured by Best [48] across a range of temperatures and wind speeds, differing from measured temperatures by <7.5% (average: 4.5%) (Fig. 3). Niche Mapper’s model denning bear in a curled position in the reported den conditions was predicted to have a steady state metabolic between the average standard metabolic rate and the lowest observed metabolic rate reported by Watts [56] (Fig. 4). When uncurled, the predicted steady state metabolic rate was greater than the average standard metabolic rate.

### Estimated Weight Loss during Simulated Fast

**Figure 5:**
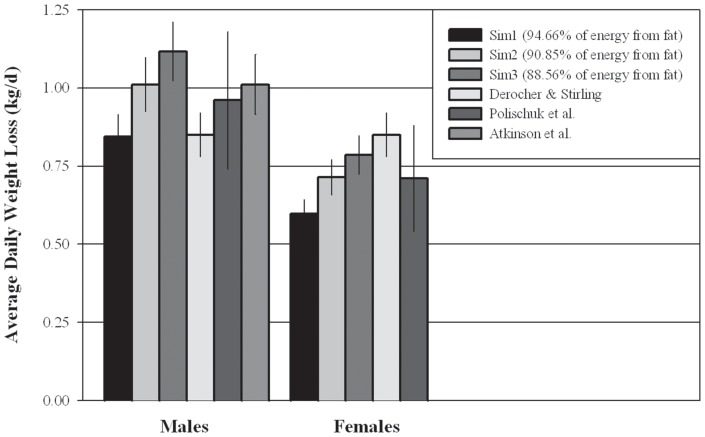
Comparison of predicted average polar bear weight loss to field measurements. Average weight losses (±S.E.) for bears of all sizes and body condition predicted by Niche Mapper simulations compared to weight losses from field studies of bears in the western Hudson Bay population during the ice-free period. The Derocher & Stirling data [67] is for adult males and females. The Polischuk et al. [60] and Atkinson et al. study [59] male data presented include both adult and subadult males, and for Polischuk et al., females with yearlings.

Simulations in which bears used higher percentages of fat for energetic demands (e.g., Sim1; 94.56%) lost less weight than those using lower percentages of fat (Fig. 5). For males, Sim1 predicted average weight loss lower than the average weight loss in two of the three studies reporting weight loss, and Sim3 (88.56% fat usage) predicted weight losses greater than all three of the reported weight losses (Fig. 5). For females, Sim1 predicted weight losses lower than published studies, while the other two simulations predicted weight losses in between the two published studies.

**Figure 6:**
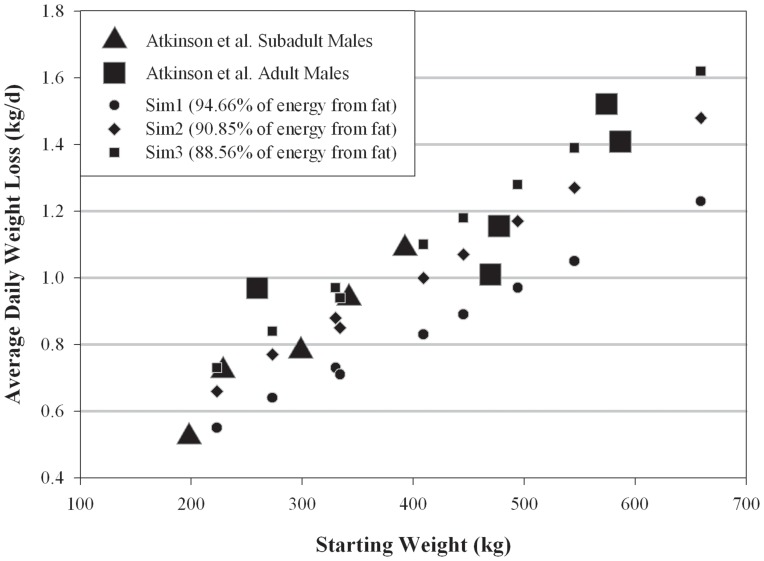
Comparing predicted mass-specific polar bear weight loss during a seasonal fast to field measurements. Weight-specific average weight losses for males predicted by Niche Mapper compared to weight-specific mass losses male bears in the western Hudson Bay population, reported in Atkinson et al. [59].

Mass-specific male weight losses predicted by Sim2 and Sim3 were comparable to weight losses reported by Atkinson et al. [59], the only study reporting such data, while Sim1 consistently predicted lower weight losses (Fig. 6).

### 120 Day Fast

Using Sim2, the intermediate weight loss simulation model assuming that fat reserves supplied 90.85% of energy needs, no male bears starting the fast in poor body condition were predicted to survive a 120 fast beginning July 29. The two larger size females in poor initial condition were expected to survive (Table 3).

**Table 3:** Percentage of initial energy reserves remaining after simulated 120 day and 180 day fasts.

		120 Day Fast			180 Day Fast		
					(1977–2006 temperatures/3°C temperature increase)		
	BodyLength (m)	PoorCondition	AverageCondition	ExcellentCondition	PoorCondition	AverageCondition	ExcellentCondition
Females	1.85	–	50.1	60.8	−/−	28.4/29.6	45.1/45.1
	2.00	6.1	52.5	62.6	−/−	32.6/33.0	47.4/47.5
	2.10	11.0	53.7	63.7	−/−	34.7/34.8	48.8/49.0
Males	2.15	–	33.0	47.4	−/−	11.9/13.2	32.0/31.8
	2.30	–	35.8	49.4	−/−	16.3/17.0	34.4/34.3
	2.45	–	38.1	51.4	−/−	19.7/19.8	36.7/36.7

Simulated 120 day fasts began July 29, and 180 day fasts began June 11. Bears of different lengths and initial body conditions (poor, average, excellent) were modeled with fat reserves assumed to supply 90.85% of energetic demands during the fast.

All average and excellent condition bears were predicted to survive, with 33–64% of their initial energy stores remaining (Table 3). The estimated minimum energy density to survive the simulated 120 day fast was 8.5 MJ/kg and 10.2 MJ/kg for males and females, respectively.

The largest bears (i.e., those with longer bodies and/or in excellent initial body condition) needed to pant for 0.8 to 7.5% of the hours in order for a heat balance to be maintained at the target metabolic rate (Table 4). All hours for which panting was needed occurred in the simulated fast’s first two weeks. Even with panting there were four hours (0.1% of all simulation hours) during which Niche Mapper could not reach a heat balance at the target metabolic rate for 210 cm long females in excellent initial condition.

**Table 4:** Percentage of hours Niche Mapper predicted panting would be required for polar bears to maintain a minimum metabolic rate during different fasting scenarios.

	Males			Females		
	215 cm pr/av/ex	230 cm pr/av/ex	245 cm pr/av/ex	185 cm pr/av/ex	200 cm pr/av/ex	210 cm pr/av/ex
**Scenario 1** a	−/−/2.9	−/−/4.7	−/1.1/6.4	−/−/3.9	−/0.8/6.3	−/1.1/7.5
**Scenario 2** b	−/−/2.5	−/−/4.5	−/−/7.2	−/−/3.9	−/−/7.2	−/−/9.5
**Scenario 3** c	−/1/9.4	−/2.7/12.0	−/4.4/15.1	−/1.4/11.7	−/3.6/14.6	−/4.7/16.9
**Scenario 4** d	−/−/−	−/−/−	−/−/1.4	−/−/−	−/−/1.3	−/−/3.6
**Scenario 5** e	−/−/2.4	−/−/5.0	−/−/8.5	−/−/4.3	−/−/8.4	−/−/11.6

Bears of different body lengths and initial body condition (poor, average, excellent) were modeled with a target metabolic rate of 2.66*mass^0.78^ W (adapted from [26]) for every hour of the day during the fast. Panting was initiated if Niche Mapper could not reach a heat balance with a metabolic rate of at least 95% of the target metabolic rate.

The different fast scenarios are as follows:

a 120 day fast beginning July 29; 1977–2006 temperatures; no shade available.

b 180 day fast beginning June 11; 1977–2006 temperatures; no shade available.

c 180 day fast beginning June 11; +3°C temperatures; no shade available.

d 180 day fast beginning June 11; 1977–2006 temperatures, shade available.

e 180 day fast beginning June 11, +3°C temperatures; shade available.

**Figure 7:**
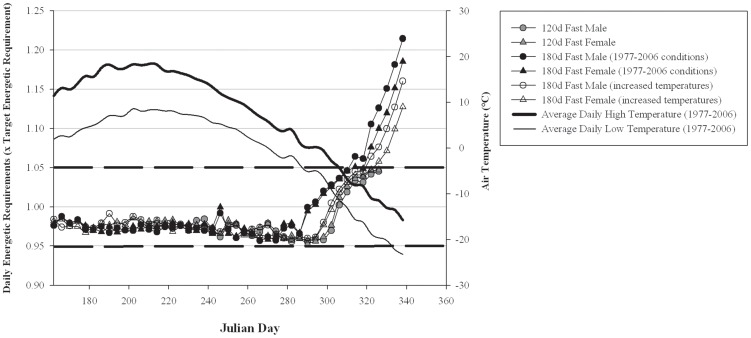
Estimated total daily metabolic requirements for fasting polar bears. Estimated total daily metabolic requirements relative to the target daily metabolic rate (55*mass^0.78 ^kcal/d; from [26]) for average sized male and female bears (body lengths of 2.3 m and 2.0 m, respectively) during the current ice-free period in the western Hudson Bay, Canada. The dashed lines show the range of values Niche Mapper would accept; values outside the dashed lines indicate that Niche Mapper exhausted thermoregulatory options before reaching a heat balance at ±5% of the target metabolic rate. These results assume that fat is used to supply 90.85% of energy reserves during the fast.

Daily energetic demands per unit body mass remained within the ±5% range from the target metabolic rate accepted by Niche Mapper for the duration of the fast for bears with average and excellent initial body condition (Fig. 7). Towards the end of their respective survival period, bears with poor initial body condition were predicted to have increasing metabolic demands in the colder temperatures. Daily metabolic requirements for average length females and males in poor initial condition began to exceed 105% of the target requirements on day 80 and 88 of the fast, respectively.

### 180 Day Fast

Using Sim2, the intermediate weight loss simulation model assuming that fat reserves supplied 90.85% of energy needs, no bears in poor initial condition were predicted to survive a 180 day fast starting on June 11 in either the 1997–2006 temperature conditions or the increased temperature conditions. All bears with average or excellent initial body conditions were still predicted to survive under both temperature conditions with 11.9 to 49% of their initial energy reserves remaining (Table 3).

Under 1977–2006 average temperature conditions the estimated minimum energy density for males and females to survive the simulated 180 day fast is 12.1 and 14.6 MJ/kg, respectively. With a 3°C temperature increase, the estimated minimum energy density for males and females to survive the simulated 180 day fast was reduced to 11.9 and 14.4 MJ/kg, respectively.

Compared to a 120 day fast starting on July 29, under 1977–2006 average temperature conditions, the hours panting was required to reach a heat balance at the target metabolic rate remained the same or declined (Table 4). With a 3°C temperature increase, panting rates more than doubled compared to the 120 day fast, and more classes of bears needed to pant (Table 4). Nearly 60% of all the predicted panting hours occurred in July, and all of the panting hours occurred between the start of the fast and the end of August. If the bears were able to seek 100% shade, panting hours were substantially reduced in both temperature conditions (Table 4).

Under 1977–2006 average temperature conditions, panting was always sufficient to allow a heat balance to be obtained during a 180 day fast beginning June 11. Under increased temperature conditions, panting was insufficient for females in excellent initial condition to reach a heat balance at the target metabolic rate for 0.2–3.5% of simulation hours (all in July and early August). Similarly 230 cm and 245 cm males in excellent initial body condition could not reach a heat balance even after panting for 0.1 and 1.7% of simulation hours, respectively. If bears were able to seek 100% shade under the increased temperature scenario, a heat balance could be obtained for all hours for all model bears.

In contrast to the 120 day fast, bears with average initial body condition were unable to keep daily energetic demands per unit body mass within the ±5% range from the target metabolic rate accepted by Niche Mapper for the duration of the 180 day fast under either temperature scenario (Fig. 7). Average initial condition bears of both sexes began to exceed 105% of expected daily energetic expenditure on fasting day 152 under 1977–2006 temperature conditions and on fasting day 160 (females) or 164 (males) under increased temperatures (Fig. 7).

Females in poor initial condition began to exceed 105% of target energetic expenditure during the last 56 days of their 132 day survival period in the 1977–2006 conditions and during the last 24 of 136 survival days under increased temperature conditions. Males in poor initial condition began to exceed 105% of target energetic expenditure in the last 28 days for their 108 day survival period in the 1977–2006 conditions, but did not exceed the target requirements before starving after 110 days under increased temperature conditions. Bears with excellent initial body condition did not need to increase metabolic expenditure at the end of the fast in either temperature scenario.

## Discussion

### Model Testing

Niche Mapper’s ability to closely match surface areas and torso diameters indicate that our allometry inputs are reasonable and that Niche Mapper is able to realistically represent and differentiate between different sized polar bears. The torso circumference measurements were higher than the axillary girth expected from equations derived from measurements on live bears. This is not surprising since Niche Mapper values are expected to be slightly higher because the cylindrical torso shape does not account the upwards slope of a bear’s torso near the armpits, where axillary girth measurements were taken in the field studies. However, this slight overestimate is only for one part of the torso, and we suspect that our torso calculations are likely reasonable approximations of average torso girth on live bears when the belly and rest of the torso is included.

Similarly, Niche Mapper’s ability to calculate similar fur temperatures and metabolic rates to live bears using its heat balance approach provides support for our biophysical inputs and accuracy of model function. Niche Mapper was unable to exactly match the measured average metabolic rate for a polar bear in simulated denning conditions. However, the curled and uncurled postures bracket, and are within the measured error bounds of, the average metabolic rate.

We recognize that this testing is far from comprehensive, and detailed metabolic studies of polar bears in different environmental conditions would be for further validation. However these comparisons to the limited relevant data available in the literature support our input choices and provide preliminary indications that Niche Mapper is able to model polar bear physiology well enough to model simulated fasting.

### Weight Loss Comparison

Overall, the predicted weight losses for both males and females are comparable to actual weight losses reported from field studies [59], [60], [67] of the population we modeled. Sim1, which tended to predict lower weight losses than those reported, based its energetic contributions of fat (94.66%) on a study of denning and reproducing bears [55]. Sim2 and Sim3 based the relative energetic contributions of fat (90.85% and 88.56%, respectively) on studies of non-denning bears [59], [60], and thus it is not surprising that weight loss results from these simulations are closer to reported weight losses for non-denning bears.

Regarding female weight loss, the Derocher and Stirling study [67] does not specify whether any of the females included in their sampling of “adult females” included bears with potential additional reproductive costs, which may explain the higher weight loss reported for females from that study. The Polischuk et al. female weight loss shown in Fig. 6 is for females with yearlings, and thus any additional reproductive costs were likely minimal.

Despite some uncertainty in making direct comparisons, the overlap between Niche Mapper simulation results– particularly with the intermediate fat usage simulation – and the reported weight losses suggests that our range of bear sizes, target metabolic rate, and proportional use of fat for energetic requirements reasonably reflect reality in the Western Hudson Bay population.

### Target Metabolic Rate

Using its steady state heat balance approach, Niche Mapper was able to meet target daily metabolic requirements (±5%) for the entire duration of a 120 day fast for bears in average and excellent initial body condition. Furthermore, Niche Mapper was able to reach a heat balance at ≥95% of the target metabolic rate without resorting to panting for the vast majority of the simulated hours (Table 4). Assuming that the average polar bear has not historically been thermally stressed during the onshore period, Niche Mapper’s ability to reach the target metabolic rate for the duration of the fast for these bears indicates that it is able to realistically model polar bear energetics and interactions with the environment.

As mentioned earlier, the metabolic rate of polar bears fasting on shore is uncertain. Under simulated denning conditions, Watts et al. [56] found resting polar bear metabolic rates averaged 73% of that expected by the mouse-to-elephant curve. When using this as a target metabolic rate, Niche Mapper predicted weight losses of 0.66–0.88 kg/d and 0.48–0.63 kg/d for males and females, respectively; these values range lower than those reported in the literature.

Using a dynamic energy budget approach, Molnar et al. [34], predicted average male metabolic rate was 107% of that expected by the mouse-to-elephant curve. Using this as a target metabolic rate, Niche Mapper predicted weight losses of 0.93 to 1.22 kg/d, which range higher than the weight losses reported in the literature. The fact that the target metabolic rate we used in our simulations provided the closest match to reported weight losses is not surprising because that rate was derived using the observed weight losses [26]. However, the closer match to published weight losses that it provides justifies its use in our simulations compared to the other potential options.

### Predicted Male Survivorship

The predicted minimum energy densities for males to survive fasts corresponds to a <3% mortality rate for a 120 fast, as estimated from Figure 3 in Molnár et al. [25]. We have no similar distribution of female energy densities with which to provide predictions about female survivorship. Niche Mapper further predicted ∼18% mortality for a 180 day fast, under 1997–2006 temperature conditions. This prediction dropped to ∼15% mortality under increased temperature conditions due to reduced thermoregulatory demands at the end of the fast. These mortality predictions are similar to those from Robbins et al.’s estimate for a 5.4 month fast (up to 16%; [26]), but lower than those predicted by Molnar et al. for a 180 day fast [25]. The Molnár et al. study predicted a minimum energy density of ∼9.25–10.15 MJ/kg for a 120 day fast (3–6% mortality for resting and active simulations, respectively) and ∼12.8–13.8 MJ/kg for a 180 day fast (28–48% mortality).

The difference in minimum energy density required for survival predicted here and Molnár et al. study appears to be mostly explained by the different metabolic rates used. The resting metabolic rate in the Molnár study averaged ∼107% of expected BMR (see [34]). When using this as the target metabolic rate, Niche Mapper predicted a minimum energy density of 9.3 MJ/kg (∼3% mortality) for a 120 day fast and 13.2 MJ/kg (∼35% mortality) for a 180 day fast, similar to the Molnár et al.’s resting predictions. The 180 day fast prediction is ∼7% higher than Molnár et al.’s prediction likely due to increased thermoregulatory costs that Niche Mapper predicts at the end of the180 fast.

As a final point of comparison, if the target metabolic rate was 73% of the average metabolic rate for three bears in simulated denning conditions [68], Niche Mapper predicts the minimum energy density for males to survive a 180 day fast to be 9.5 MJ/kg (∼8% mortality). This again underscores the importance of understanding resting metabolic rates of polar bears onshore.

### Impact of Environmental Conditions on Polar Bear Energetic Predictions

Niche Mapper’s metabolic predictions suggest that polar bears are not entirely free of thermal constraints while ashore during the ice-free season, and that different classes of bears are affected during different times of the fast. During the 120 day fast beginning July 29, bears starting out the fast in poor body condition needed a metabolic rate greater than the target value in order to stay in thermal steady state with the environment during the colder days of its survival period. Bears in average and excellent initial condition did not need to elevate metabolic rates in the same manner because they had more of their insulative fat reserves remaining when the temperatures began to drop.

However, the potential impact of the environment on average and excellent initial condition bears is evident when extending the fast from 120 days (beginning July 29) to 180 days (beginning June 11). Average initial condition bears would then enter the colder temperatures at the end of the fast with less insulation, having relied on fat reserves for a longer period of time. As a result, under both 180 day fast temperature conditions simulated, average condition bears were predicted to need to elevate metabolic rates to reach a thermal balance, even on Julian days when an elevated metabolic rate was not needed during the 120 day fast (Fig. 7).

Excellent initial condition bears never had daily metabolic expenditure deviate more than 5% above the target rate, even during the coldest days of the fast. However, Niche Mapper predicted that these bears would need to increase the amount of active thermoregulation (i.e., panting) during the warmer days of the fast in order to maintain their metabolic rate, particularly when the increase in ice-free period was assumed to be accompanied by a 3°C increase in temperature. Historically, the hottest days in the area occur in July, and by extending the fast to include all of July, bears were exposed to more of these hotter days. Under the increased temperature scenario, at times some excellent condition bears could not reach a thermal balance even after panting, which indicates the need for behavioral thermoregulation such as swimming, which is likely a more energetically expensive thermoregulatory mechanism than panting.

Panting is associated with metabolic rate increases due to the muscle activity required for the increased ventilation rate (e.g., [69]). The current version of Niche Mapper does not account for this, and thus thermoregulatory benefits from panting are energetically “free”. Therefore, the energetic expenditure predictions for the bear classes for which panting was predicted are likely underestimates, particularly in the increased temperature simulations. However, assuming a 25% metabolic rate increase when panting [69], survival estimates are unaffected since poorer condition bears (including the poorest condition bear predicted to survive the 180 day fast) were not predicted to require panting, and those bears that did need to pant had more than enough reserves remaining to account for the additional cost of panting.

It should also be noted that Niche Mapper assumes a particular order of thermoregulatory options, with panting only occurring after all other thermoregulatory options have been exhausted. It could be the case that bears may engage in energetically expensive thermoregulation such as panting or swimming before having core temperatures rise as a result of heat storage. This would even further increase energy expenditure above that predicted by Niche Mapper, and may affect even poorer condition bears, reducing their survival period.

Furthermore, the target metabolic rate used in our simulations is an average of all energetic expenditure during a fasting period, including any activity [26]. Thus, the true resting rate is likely lower than the target rate used here, and thus thermoregulation to avoid overheating would not be triggered as quickly as it is in our simulations. However, as noted earlier, fasting bears spend over 90% of their time resting, and thus the “true” resting rate is likely close to the target rate we used, particularly since the model allows for 5% variation from the target rate before engaging in thermoregulation.

### Advantages of Niche Mapper as a Mechanistic Model

This study’s results highlight the importance of considering environmental conditions when making predictions about the polar bear energetics, particularly when attempting to make predictions with respect to a changing climate. Metabolic predictions made here suggest that bears are not entirely free of thermal constraints while ashore during the ice-free season. On the one hand, the largest bears are subject to overheating in warmer temperatures, and on the other hand, bears at the end of the fast with depleted fat reserves may not be able to maintain the target resting metabolic rate that they could maintain during earlier, warmer portions of the fast. Thus, any modeling predictions that do not take into account environmental conditions run the risk of over- or underestimating polar bears’ energetic demands, as illustrated by the mortality estimates for a 180 day fast beginning June 11 decreasing from 18% to 15% with a 3°C uniform increase in daily temperatures.

These results also suggest that the impact of environmental conditions on bear energetics will depend on both the magnitude and the timing of the temperature increase. If most of the warming occurs in the summer, there will be increased metabolic requirements for thermoregulation. However, if most of the warming occurs in the winter, the energetic requirements may be minimized by reducing thermoregulatory requirements in the colder days at the end of the fast. Similarly, the impact of climate change will also depend on the degree to which daily high and low temperatures are affected.

Niche Mapper’s heat balance approach also helps to identify potentially important habitat for polar bears onshore. The increased panting rates with a 3°C temperature increase indicate that polar bears may be near the edge of their thermal neutral zone in the 1977–2006 conditions. Thus habitat selection to minimize energetic expenditure on thermoregulation will become increasingly important as daily maximum temperatures rise. Having access to water for swimming, shade for bears inland, high areas of land to be exposed to stronger winds, or riverbank caves or depressions dug down close to the permafrost may be important for preventing even resting bears from overheating (c.f. [70]).

An important limitation of this study is that the mortality estimates for the longer fast do not take into account the fact that bears moving to shore earlier in the year have less time to feed and thus are likely to be in a different body condition than bears leaving the ice later in the summer [25], [71]. While the minimum energy density necessary to survive a given fast will not be affected by this consideration, the proportion of the population that has the required minimum energy density at the start of the fast is likely to be lower than the current proportion indicated in the work by Molnár et al. [25]. Thus, the future mortality predictions presented are likely conservative.

Validation of this model is also currently limited by lack of empirical data. As discussed earlier, the resting metabolic rates for fasting polar bears onshore during the ice-free season is not well understood. Metabolic rate is the primary driver of weight loss, survival, and need for thermoregulation. Thus, a better understanding would allow for more confident predictions. Additionally, three studies have reported different body reserve usages by polar bears. These different uses of body fat and lean body mass for energy requirements lead to distinctly different weight loss patterns (Fig. 5), and we are basing mortality predictions on the intermediate weight loss simulation (Sim2). However, if bears use body fat as reported in Atkinson and Ramsay [58] (modeled in Sim1), we would have to increase the target metabolic levels to match the reported weight losses. This increased energy expenditure would then result in higher mortality rates.

Model predictions could also be improved with more precise site-specific environmental data. For example, as the sensitivity analyses in the Supporting Information illustrate, wind speeds have a large impact on steady state metabolic rates, particularly in colder temperatures. In this study we used the average daily wind speed as both the maximum and minimum. However, if overnight wind speeds tended to be lower, thermoregulatory costs during the cold nights at the end of the fast may be reduced. Similarly, higher wind speeds during the day in the fast’s warmer days may reduce the need for active thermoregulation to prevent overheating.

However, despite these limitations, Niche Mapper can still produce testable hypotheses about polar bear interactions with the environment and the need for additional energetic expenditure for thermoregulation under different fasting scenarios. Model predictions could be verified through comparisons to observable thermoregulatory behavior like panting, swimming, or shade seeking. Furthermore the model can be applied to polar bear populations that do not currently experience a seasonal ice-free period, but may sometime in the future. Application of the model to such populations could aid in both survivorship predictions as well as ensuring that bears have access to terrestrial habitats needed to minimize potential thermoregulatory costs.

Since generic biophysical models like Niche Mapper rely only on interactions with user-specified microclimate conditions and animal physiological, morphological, and behavioral properties, users can place virtual animals into novel environmental conditions and obtain reliable estimates of energetic requirements. This information could aid predictions of the impact of climate change on polar bear survival, distribution, body size, and reproductive success, (e.g. Kearney et al. [72]), all of which are critical factors when developing management plans. By explicitly linking polar bear energetics to environmental conditions, Niche Mapper can thus play in important role in helping to inform demographic projection modeling under uncertain climate change.

## Supporting Information

Figure S1 **Effect of cloud cover on a simulated polar bear simulated on Julian Day 162.** Thermoregulatory options (see Text S1) were disabled to clearly illustrate the effect of cloud cover. Increasing cloud cover resulted in less incoming solar radiation available to the bears during daylight hours, increasing the steady state metabolic rate. During nighttime hours, increasing cloud cover decreased the steady state metabolic rate because bears when clouds are present animals have radiant heat exchange with the clouds rather than the clear sky, which has a much lower temperature.
(TIF)

Figure S2 **Sensitivity of Niche Mapper to various model inputs when modeling a furred cylinder: a) wind speed; b) fur depth; c) flesh thermal conductivity; d) fur thermal conductivity; e) core temperature; f) cylinder circumference.** A 1 m long cylinder with a flesh radius of 0.3 m and 0.04 m thick fur was used in these analyses. See Table S1 for inputs used in each sensitivity analysis. Notes: A 5–10% increase or decrease in cylinder circumference corresponds to ∼5–10% increase or decrease in surface area. Relative humidity (analysis not shown) did not meaningfully affect metabolic rate calculations (metabolic rate predictions between 0% and 100% relative humidity varied by an average of 1.5% from −35°C to +35°C range of temperatures).
(TIF)

Figure S3 **Polar bears as modeled by Niche Mapper.** Whole animals are broken down into a series of cylinders (or truncated cone, in the case of the head) representing different body parts, as illustrated in the figure. Measurements (in m) provided are for male and female bears of average length (2.3 m and 2.0 m, respectively) in average body condition (total mass = 2.25× structural mass), with fur depths of 10, 40, 50 and 35 cm for the head, neck, torso and legs, respectively.
(TIF)

Figure S4 **Metabolic chamber simulations of an average sized female polar bear (200 cm long) in various body conditions (total body mass 1.5 (a), 2.25 (b), and 3.0 (c) times structural mass, representing poor, average and excellent body condition, respectively) in both summer and winter fur coat.** The trends in the outputs (i.e., the temperatures at which bears in various body conditions cannot thermoregulate to maintain metabolic rate within certain percentages of expected basal metabolic rate from the mouse to elephant curve) are representative of all body lengths modeled for both sexes. The model bears were able to thermoregulate by varying flesh thermal conductivity (0.5–2.8 W/mC), varying core temperature (36–39°C), panting, and adjusting body posture by curling up with legs tucked into the torso to minimize heat loss. A wind speed of 4 m/s and relative humidity of 5% was used in all simulations.
(TIF)

Figure S5 **Impact of wind (4 m/s) on metabolic rates predicted for Niche Mapper for an average sized female polar bear in summer fur coat compared to rates predicted under windless conditions.**
(TIF)

Table S1 **Parameter inputs used in the sensitivity analyses presented in Fig. S2.**
(DOC)

Text S1 **Supporting Information Heat Balance Solution Procedure.**
(PDF)
