# Supplementary figures and images for: Simulating Polar Bear Energetics during a Seasonal Fast Using a Mechanistic Model
Source: PLoS One. 2013 Sep 3;8(9):e72863. doi: 10.1371/journal.pone.0072863 (PMC3760880; doi:10.1371/journal.pone.0072863)

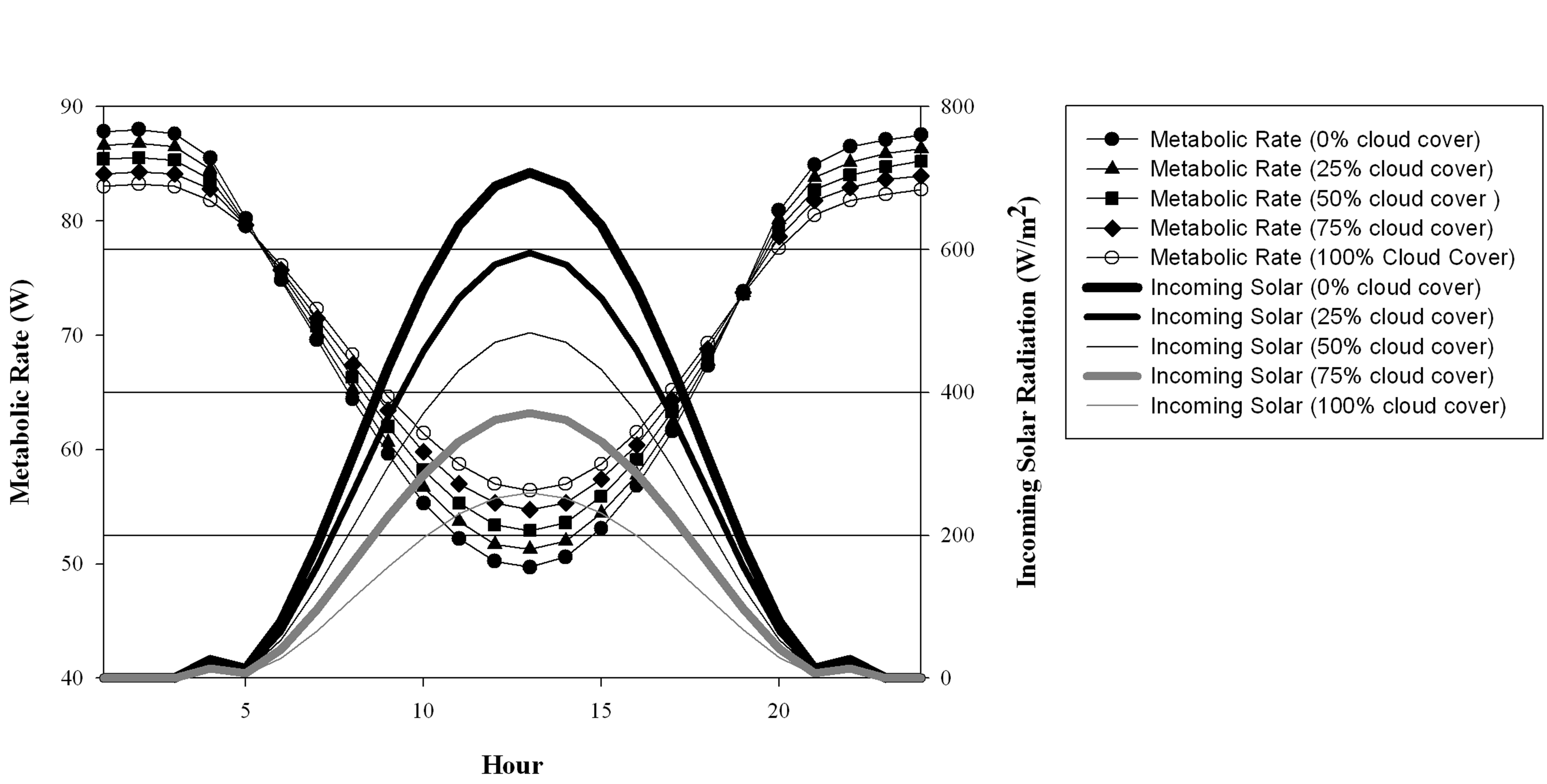

Supplement: Figure S1 — Effect of cloud cover on a simulated polar bear simulated on Julian Day 162. Thermoregulatory options (see Text S1) were disabled to clearly illustrate the effect of cloud cover. Increasing cloud cover resulted in less incoming solar radiation available to the bears during daylight hours, increasing the steady state metabolic rate. During nighttime hours, increasing cloud cover decreased the steady state metabolic rate because bears when clouds are present animals have radiant heat exchange with the clouds rather than the clear sky, which has a much lower temperature. (TIF) [file pone.0072863.s001.tif]

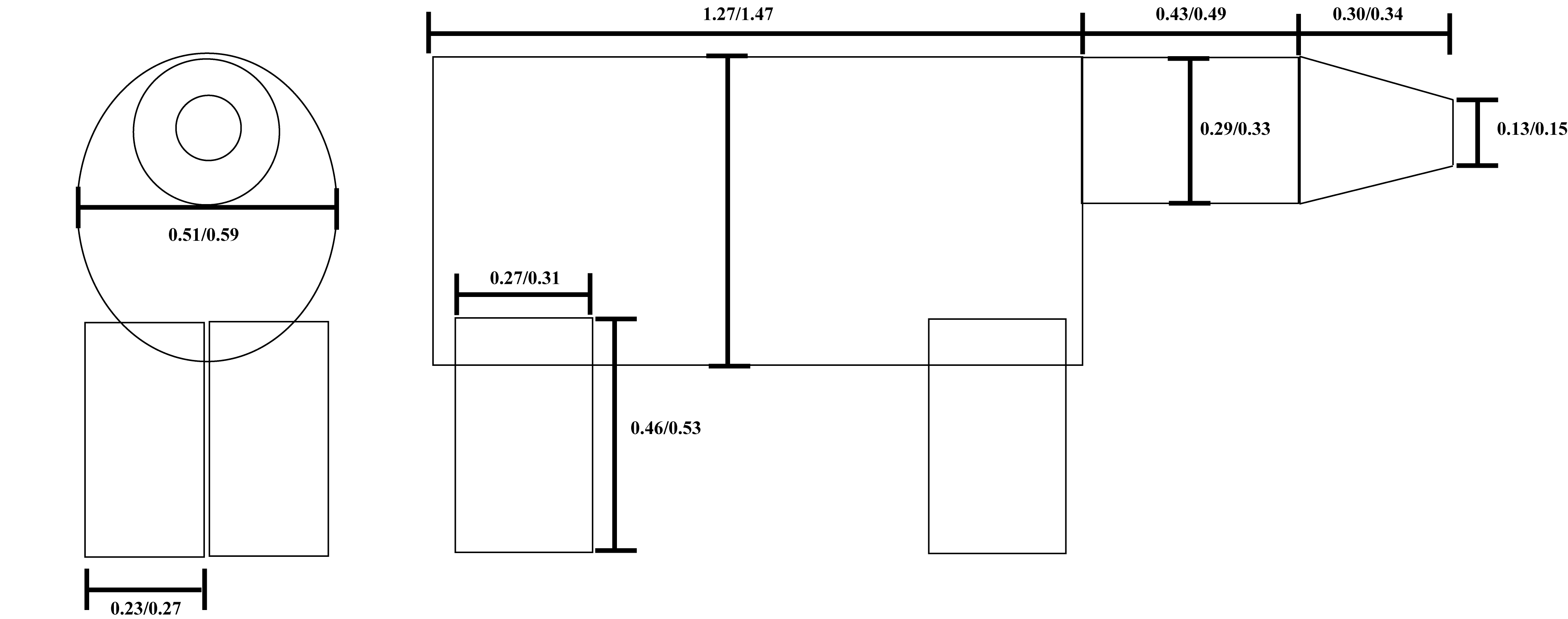

Supplement: Figure S3 — Polar bears as modeled by Niche Mapper. Whole animals are broken down into a series of cylinders (or truncated cone, in the case of the head) representing different body parts, as illustrated in the figure. Measurements (in m) provided are for male and female bears of average length (2.3 m and 2.0 m, respectively) in average body condition (total mass = 2.25× structural mass), with fur depths of 10, 40, 50 and 35 cm for the head, neck, torso and legs, respectively. (TIF) [file pone.0072863.s003.tif]

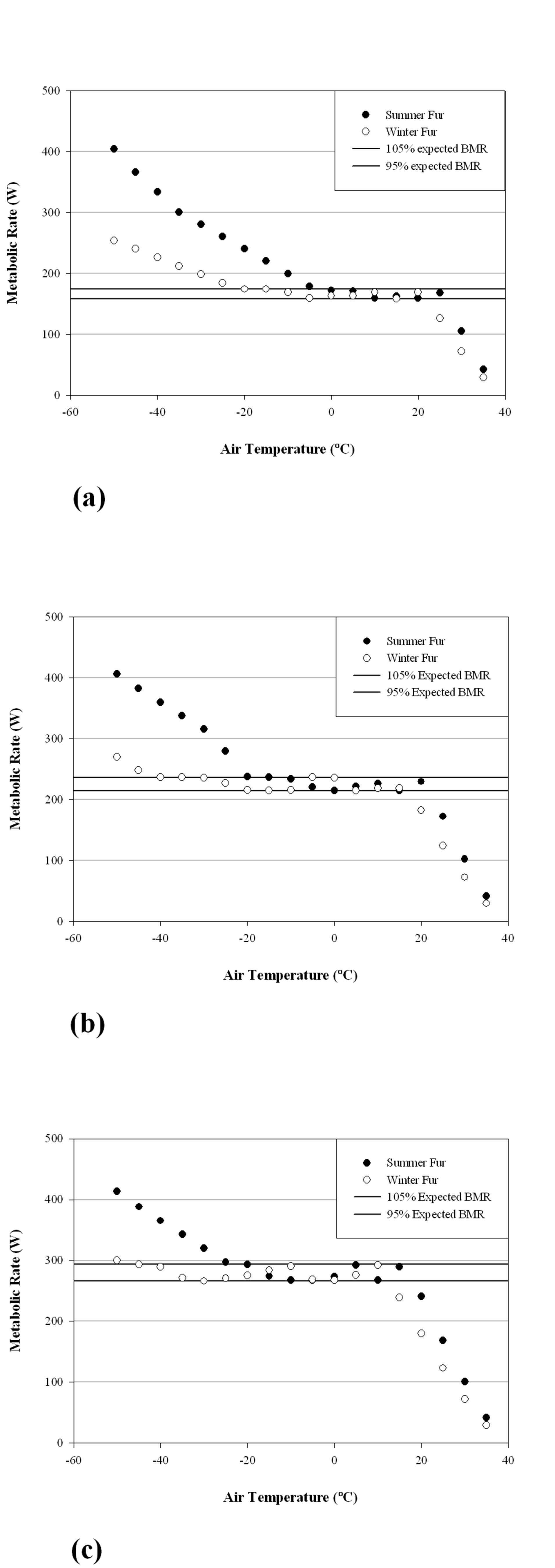

Supplement: Figure S4 — Metabolic chamber simulations of an average sized female polar bear (200 cm long) in various body conditions (total body mass 1.5 (a), 2.25 (b), and 3.0 (c) times structural mass, representing poor, average and excellent body condition, respectively) in both summer and winter fur coat. The trends in the outputs (i.e., the temperatures at which bears in various body conditions cannot thermoregulate to maintain metabolic rate within certain percentages of expected basal metabolic rate from the mouse to elephant curve) are representative of all body lengths modeled for both sexes. The model bears were able to thermoregulate by varying flesh thermal conductivity (0.5–2.8 W/mC), varying core temperature (36–39°C), panting, and adjusting body posture by curling up with legs tucked into the torso to minimize heat loss. A wind speed of 4 m/s and relative humidity of 5% was used in all simulations. (TIF) [file pone.0072863.s004.tif]

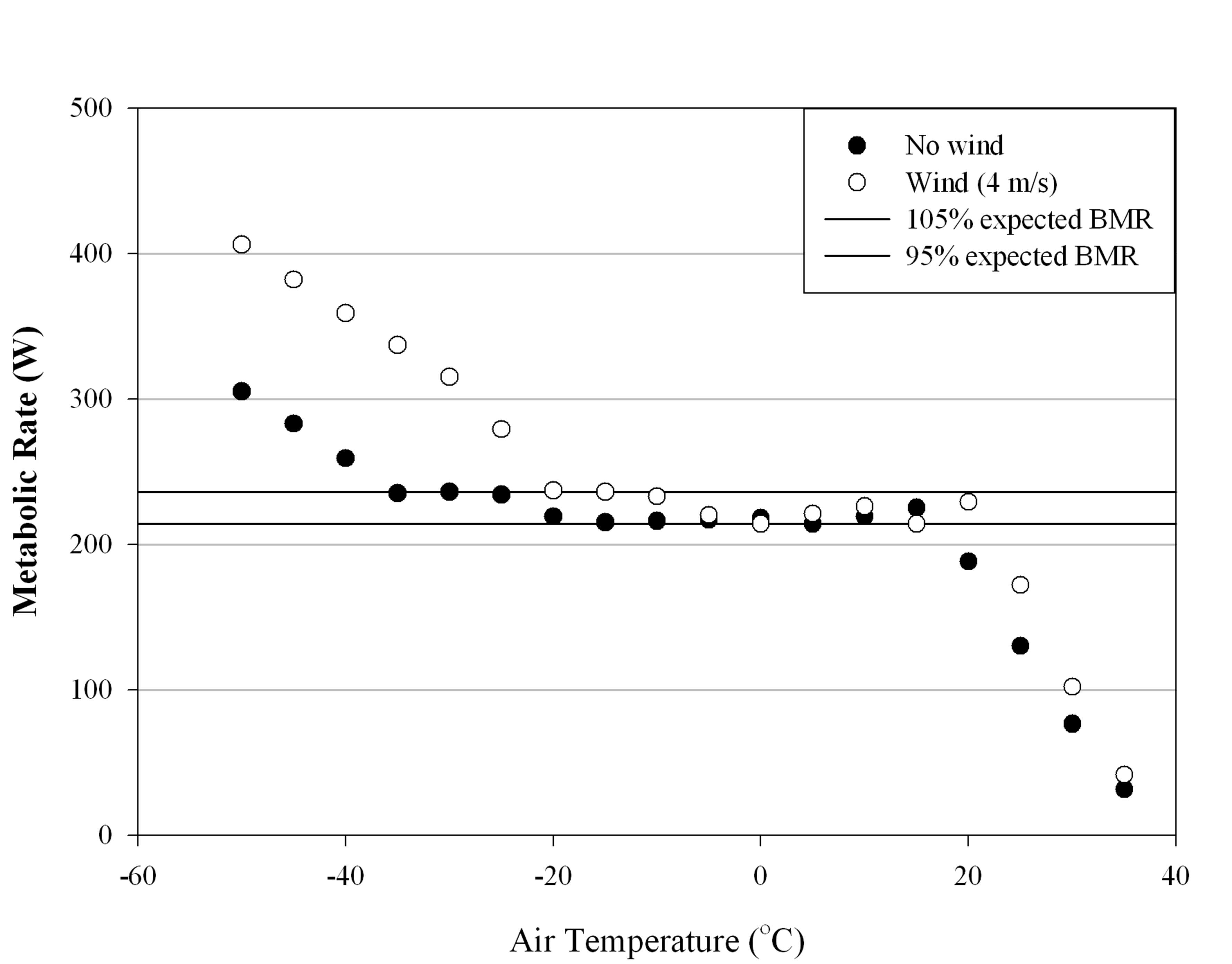

Supplement: Figure S5 — Impact of wind (4 m/s) on metabolic rates predicted for Niche Mapper for an average sized female polar bear in summer fur coat compared to rates predicted under windless conditions. (TIF) [file pone.0072863.s005.tif]
