# Supplementary material for: Simulating Polar Bear Energetics during a Seasonal Fast Using a Mechanistic Model
Source: PLoS One. 2013 Sep 3;8(9):e72863. doi: 10.1371/journal.pone.0072863 (PMC3760880; doi:10.1371/journal.pone.0072863)
Supplement: Table S1 — Parameter inputs used in the sensitivity analyses presented in Fig. S2. (DOC) [file pone.0072863.s006.doc]

**Table S1.** Parameter inputs used in the sensitivity analyses presented in Fig. S2.

| **Sensitivity Analysis** | **Cylinder Length (m)** | **Fur Depth (m)** | **Circumference (m)** | **Core Temperature (°C)** | **Fur thermal conductivity (W/mC)** | **Flesh thermal conductivity (W/mC)** | **Wind Speed (m/s)** | **Relative Humidity (%)** |
| --- | --- | --- | --- | --- | --- | --- | --- | --- |
| Wind Speed | 1.0 | 0.04 | 0.94 | 37.0 | 0.063 | 1.0 | Variable | 5.0 |
| Flesh thermal conductivity | 1.0 | 0.04 | 0.94 | 37.0 | 0.063 | Variable | 4.0 | 5.0 |
| Fur thermal conductivity | 1.0 | 0.04 | 0.94 | 37.0 | Variable | 1.0 | 4.0 | 5.0 |
| Core temperature | 1.0 | 0.04 | 0.94 | Variable | 0.063 | 1.0 | 4.0 | 5.0 |
| Circumference | 1.0 | 0.04 | Variable | 37.0 | 0.063 | 1.0 | 4.0 | 5.0 |
| Fur Depth | 1.0 | Variable | 0.94 | 37.0 | 0.063 | 1.0 | 4.0 | 5.0 |
