# Supplementary material for: Simulating Polar Bear Energetics during a Seasonal Fast Using a Mechanistic Model
Source: PLoS One. 2013 Sep 3;8(9):e72863. doi: 10.1371/journal.pone.0072863 (PMC3760880; doi:10.1371/journal.pone.0072863)
Supplement: Text S1 — Supporting Information Heat Balance Solution Procedure. (PDF) [file pone.0072863.s007.pdf]

1 Heat Balance Solution for distributed heat generation in a cylinder covered in fur

2 I. Overview and overall heat balance at steady state:

Endothermic animals endogenously produce heat and exchange heat with their environment through convection, evaporation and absorption and emission of radiant energy. For an endotherm to maintain its core temperature, it cannot endogenously generate more or less heat than it is exchanging with its surroundings. Thus, the heat balance for a furred endotherm maintaining its core temperature in its surroundings can be expressed as follows:

$$Q_{gen,net} - Q_{evap} = Q_{fur} = Q_{env} \tag{1}$$

where

$$Q_{gen,net} = Q_{gen} - Q_{resp} \tag{2}$$

and

$$Q_{env} = Q_{rad} + Q_{conv} - Q_{sol} \tag{3}$$

Q_{gen} is the heat generated by metabolic processes, Q_{resp} is the heat loss by evaporation from the respiratory tract, Q_{evap} is evaporation from the skin, Q_{rad} is the net infrared thermal radiation between the animal and its environment, Q_{conv} is the heat loss by convection and Q_{sol} is the heat absorbed from solar radiation.

That is, metabolic heat production in the body (Q_{gen}), less evaporative heat loss from the respiratory system and the skin, must equal heat flow through the fur (Q_{fur}) and the net heat flux with the outside environment for the animal to be in thermal steady state with its environment. Deviations from the equality will result a net heat loss or heat gain, rendering the animal unable to maintain its body temperature.

This heat balance equation allows for a calculation of what metabolic rate is needed for an animal to maintain its body temperature in its particular microclimate conditions. As illustrated in the solution below, the heat flux through each layer (core to skin; skin to fur-air interface; fur-air interface to environment) is dependent on the temperature gradient existing in each layer, the thermal conductivity of each layer, and the shape and dimensions of each layer.

However, the only known temperatures for the model animals are the core temperature and the surrounding air temperature. We must calculate the skin temperature and the fur-air interface temperature in order to solve the heat balance and determine what metabolic rate is required for the animal to maintain its body temperature in its current environmental conditions.

The solution described below shows how this is performed in Niche Mapper. It is an update to previous the previous heat balance calculation done by Niche Mapper (see, e.g., Porter et al. 1994, 2000, 2002, 2006), in which the heat flux in each layer (body, fur, and environment) were calculated individually, working from the core to the environment. This created a disconnect between internal and external factors affecting the heat balance. The modification described below ensures that the skin and fur-air interface temperature boundary conditions are calculated simultaneously as a function of internal (e.g., core temperature, metabolic heat production) and external (i.e., environmental) conditions. This allows a more precise, accurate and efficient solution. The previous solution procedure always carried a degree of error, while this updated solution requires an exact solution.

II. Assumptions

1. All solar is absorbed at the coat surface (i.e., does not penetrate into fur layer) (Turnpenny et al. 2000, Mount & Brown 1982, Stafford Smith et al. 1985).

2. Evaporative heat loss takes place at the skin surface and thus this component of $Q_{gen,net}$ does not make it to the fur layer.

3.The remainder of net metabolic heat generation that is not lost through evaporation must be conducted through the fur/feather layer and then be convected or radiated away from the fur surface.

4.There is negligible free convection within the fur layer (Davis and Birkebak 1975).

5. Respiratory heat loss does not affect skin or fur-air temperature.

III. Mechanism Equations

The heat generating cylinder solution with an insulating layer is (Bird et al., 2002)

$$Q_{gen,net} = \frac{(T_c - T_s)}{\frac{R_G^2}{4k_G V_G} + \frac{R_G^2}{2k_I V_G} \ln\left(\frac{R_S}{R_G}\right)} \tag{4}$$

where T_c means core temperature and T_s means skin temperature. The subscripts G and I mean (flesh) heat Generating tissue and (fat) Insulating tissue respectively, k is the thermal conductivity $(\frac{W}{mC})$. R is the radial dimension (m).

The hollow cylinder solution for fur with no internal solar heat absorption is (Bird et al., 2002)

$$Q_{fur} = \frac{2\pi k_f L (T_S - T_{FA})}{\ln\left(\frac{R_L}{R_s}\right)} \tag{5}$$

where k_f is the fur thermal conductivity, T_{FA}means fur-air interface temperature and R_L is the radial distance from the center of the animal to the fur air interface. The variable, L, is the thickness of the fur.

Heat exchange with the environment by long wavelength infrared, convection and solar radiation are respectively

$$Q_{rad} = h_r A_{FA} (T_R - T_{SKY}) \tag{6}$$

where h_r is a Taylor Series expansion, $h_r = 4\sigma T_{ave}^3$, where $T_{ave} = (\frac{T_r + T_{SKY}}{2})$ and A_{FA} is the fur-air interface surface area.

Equation 5 closely approximates the exact Stefan-Boltzmann equation,

$$Q_{rad} = \sigma A_{FA} (T_R^4 - T_{SKY}^4) \tag{7}$$

, where T is in Kelvin. There is only a 1.1% error when the temperature difference between T_R and T_{SKY} is 60 ^oC.

The long wavelength emissivity, ε, in the infrared thermal equations is assumed = 1.0 to simplify the algebra presentation.

$$Q_{conv} = h_c A_{FA} (T_{FA} - T_A) \tag{8}$$

where h_c is the heat transfer coefficient $(\frac{W}{m^2C})$, usually experimentally determined and a function of the geometric shape, wind speed and fluid properties.

and

$$Q_{sol} = \alpha A_{FA} I_{sol} \tag{9}$$

where α is the fur solar absorptivity and I_{sol} is incoming solar radiation (W/m^2).

IV. Deriving two Tskin equations in the context of metabolic heat generation.

1. From the body derivations, i.e. equation 3:

$$T_s = T_c - \frac{Q_{gen,net} \cdot R_G^2}{4k_G V_G} - \frac{Q_{gen,net} \cdot R_G^2}{2k_I V_G} \cdot \ln\left(\frac{R_S}{R_G}\right) \tag{10}$$

2. From the fur derivations, i.e. from the solution of a hollow cylinder fur layer surrounding a cylinder of heat generating flesh of a certain volume (V_G) (Bird et al., 2002).

$$T_s = \left[\frac{Q_{gen,net} \cdot R_G^2}{4k_f \cdot V_G}\right] \cdot \ln\left(\frac{R_L}{R_S}\right) + T_{FA} \tag{11}$$

But, since in the fur, some of the net metabolic heat generation has been lost via evaporation, only the remainder is flowing through the fur layer:

$$T_s = \left[\frac{(Q_{gen,net} - Q_{evap}) \cdot R_G^2}{2k_f \cdot V_G}\right] \cdot \ln\left(\frac{R_L}{R_S}\right) + T_{FA} \tag{12}$$

V. Solving for $\mathbf{T_{FA}}$ in order to calculate $\mathbf{Q_{rad}}$ and $\mathbf{Q_{conv}}$.

The challenge is to do this without using T_s, which, along with $Q_{\mathbf{gen}}$ and $T_{\mathbf{FA}}$, is an unknown value.

Step 1: Setting up the balance in terms of T_{FA} :

$$Q_{fur} = Q_{rad} + Q_{conv} - Q_{sol} \tag{13}$$

Note: Q_{rad} is simplified below to consider only radiant exchange with the sky for purposes of clearer explanation of the procedure. The complete Q_{rad} will be defined at the end of this development.

Inserting the mechanism equations for heat transfer by thermal infrared radiation and convection

$$\frac{2\pi k_f L}{\ln\left(\frac{R_L}{R_S}\right)}(T_S - T_{FA}) = h_r A_{FSky}(T_R - T_{SKY}) + h_c A_{FA}(T_{FA} - T_A) - Q_{sol} \tag{14}$$

where the constant, $\frac{2\pi k_f L}{\ln\left(\frac{R_L}{R_S}\right)}$ in Q_{fur} is abbreviated as "C_f", with units W/C for algebraic simplicity.

Step 2: Eliminating T_S

- We know from the body that

$$T_s = T_c - \frac{Q_{gen,net} \cdot R_G^2}{4k_G V_G} - \frac{Q_{gen,net} \cdot R_G^2}{2k_I V_G} \cdot \ln\left(\frac{R_S}{R_G}\right) \tag{15}$$

- We also know from the steady state that

$$Q_{gen,net} - Q_{evap} = Q_{fur} \tag{16}$$

- So we can say

$$T_s = T_c - \frac{(Q_{fur} + Q_{evap}) \cdot R_G^2}{4k_G V_G} - \frac{(Q_{fur} + Q_{evap}) \cdot R_G^2}{2k_I V_G} \cdot \ln\left(\frac{R_S}{R_G}\right) \tag{17}$$

- Thus, Q_{fur} can be rewritten as: $Q_{fur} = C_f \left[T_c - \frac{(Q_{fur}+Q_{evap}) \cdot R_G^2}{4k_G V_G}\right] - \frac{(Q_{fur}+Q_{evap}) \cdot R_G^2}{2k_I V_G} \cdot \ln\left(\frac{R_S}{R_G}\right) - C_f \cdot T_{FA}$
- Expanding so that the Q_{fur} term can be factored out

$$Q_{fur} = C_f \left[\begin{array}{c} T_c - \frac{Q_{fur} \cdot R_G^2}{4k_G V_G} - \frac{Q_{evap} \cdot R_G^2}{4k_G V_G} - \frac{Q_{fur} \cdot R_G^2}{2k_I V_G} \cdot \ln\left(\frac{R_S}{R_G}\right) \\ - \frac{Q_{evap} \cdot R_G^2}{2k_I V_G} \cdot \ln\left(\frac{R_S}{R_G}\right) \end{array} \right] - C_f \cdot T_{FA} \tag{18}$$

- Expanding:yet again $Q_{fur} = C_f \cdot T_c - \frac{C_f \cdot Q_{fur} \cdot R_G^2}{4k_G V_G} - \frac{C_f \cdot Q_{evap} \cdot R_G^2}{4k_G V_G} - \frac{C_f \cdot Q_{fur} \cdot R_G^2}{2k_I V_G} \cdot \ln\left(\frac{R_S}{R_G}\right) - \frac{C_f \cdot Q_{evap} \cdot R_G^2}{2k_I V_G} \cdot \ln\left(\frac{R_S}{R_G}\right) - C_f \cdot T_{FA}$
- Collecting Q_{fur} terms, and factoring out Q_{fur}:

$$\begin{aligned} & Q_{fur} \cdot \left[1 + \frac{C_f \cdot R_G^2}{4k_G V_G} + \frac{C_f \cdot R_G^2}{2k_I V_G} \cdot \ln\left(\frac{R_S}{R_G}\right)\right] \\ = \; & C_f \cdot T_c - C_f \left[\frac{Q_{evap} \cdot R_G^2}{4k_G V_G} - \frac{Q_{evap} \cdot R_G^2}{2k_I V_G} \cdot \ln\left(\frac{R_S}{R_G}\right)\right] - C_f \cdot T_{FA} \end{aligned} \tag{19}$$

where the constant $\left[1 + \frac{C_f \cdot R_G^2}{4k_G V_G} + \frac{C_f \cdot R_G^2}{2k_I V_G} \cdot \ln\left(\frac{R_S}{R_G}\right)\right]$ is abbreviated "D_1" (unitless) to simplify the algebra and the term $\left[\frac{Q_{evap} \cdot R_G^2}{4k_G V_G} - \frac{Q_{evap} \cdot R_G^2}{2k_I V_G} \cdot \ln\left(\frac{R_S}{R_G}\right)\right]$ is abbreviated "D_2" (units = degrees C).

- Thus,

$$Q_{fur} = \frac{C_f \cdot T_c}{D_1} - \frac{C_f \cdot D_2}{D_1} - \frac{C_f \cdot T_{FA}}{D_1} = \frac{C_f}{D_1} \cdot (T_c - D_2 - T_{FA}) \tag{20}$$

- Now back to the $Q_{fur} = Q_{rad} + Q_{conv} - Q_{sol}$ heat energy balance with T_s removed: $\frac{C_f}{D_1} \cdot (T_c - D_2 - T_{FA}) = h_r \cdot A_{FSky} \cdot (T_R - T_{SKY}) + h_r \cdot A_{FGrd} \cdot (T_R - T_{GRD}) + h_c \cdot A_{FA} \cdot (T_{FA} - T_A) - Q_{sol}$

Step 3: Writing T_R in terms of T_{FA}

- $R_{rad} = R_S + [X_r \cdot (R_L - R_S)]$, where X_r = a specified depth into the fur where radiant exchange takes place (1 = at the fur surface, 0.5 = at the midpoint depth).
- Given the fur temperature profile:

$$T_f = \left[\frac{(Q_{gen,net} - Q_{evap}) \cdot R_G^2}{2k_f \cdot V_G}\right] \cdot \ln\left(\frac{R_L}{r}\right) + T_{FA} \tag{21}$$

The effective radiant temperature, T_R, is

$$T_R = \left[\frac{(Q_{gen,net} - Q_{evap}) \cdot R_G^2}{2k_f \cdot V_G}\right] \cdot \ln\left(\frac{R_L}{R_{rad}}\right) + T_{FA} \tag{22}$$

where $(Q_{gen,net} - Q_{evap}) = Q_{fur} = \frac{C_f}{D_1} \cdot (T_c - D_2 - T_{FA})$. Thus

$$T_R = \left[\frac{\frac{C_f}{D_1} \cdot (T_c - D_2 - T_{FA}) \cdot R_G^2}{2k_f \cdot V_G} \right] \cdot \ln\left(\frac{R_L}{R_{rad}}\right) + T_{FA} \tag{23}$$

where the constant, $\left[\frac{\frac{C_f}{D_1} \cdot (T_c - D_2 - T_{FA}) \cdot R_G^2}{2k_f \cdot V_G} \right] \cdot \ln\left(\frac{R_L}{R_{rad}}\right)$, is abbreviated as "$D_3$" (units = C).

- Thus: $Q_{rad} = h_r \cdot A_{FA} \cdot ((D_3 + T_{FA}) - T_{SKY})$
- Back to the fur heat energy balance, $Q_{fur} = Q_{rad} + Q_{conv} - Q_{sol}$ with T_S removed and T_r now in terms of T_{FA}:

$\frac{C_f}{D_1} \cdot (T_c - D_2 - T_{FA}) = h_r \cdot A_{FA} \cdot ((D_3 + T_{FA}) - T_{SKY}) + h_c \cdot A_{FA} \cdot (T_{FA} - T_A) - Q_{sol}$

Step 4: Expanding, rearranging and solving for T_{FA}:

$$T_{FA} = \frac{h_r \cdot A_{FA} \cdot (T_{SKY} - D_3) + h_c \cdot A_{FA} \cdot T_A + \frac{C_f \cdot (T_c - D_2)}{D_1} + Q_{sol}}{h_r \cdot A_{FA} + h_c \cdot A_{FA} + \frac{C_f}{D_1}} \tag{24}$$

VI. Solution Procedure

Step 1. Guess an initial T_{FA} to get things started since the h_c and h_r terms in the final T_{FA} calculation depend on T_{FA}. The first guess should be T_A.

Step 2. Guess an initial T_S because Q_{evap} depends on T_S and we could not find a way to remove this. Using the standard definition of T_S from the body definition would introduce another unknown ($Q_{gen,net}$), so that does not help. The first T_S guess should be T_c.

Step 3. Calculate T_{FA} from the equation in Part V, Step 4, using the T_{FA} guess to calculate the h_c and h_r terms and the T_S guess to calculate Q_{evap}.

Step 4. Adjust the T_{FA} guess until the resulting calculated T_{FA} matches the starting T_{FA} guess. NicheMapper does this by adjusting the T_{FA} starting guess to match the previously-calculated T_{FA} and then calculating another T_{FA} using equation 26. There is typically convergence within 5-10 guesses and subsequent adjustments on the starting guess.

Step 5. Using the T_{FA} calculation from Step 4, calculate Q_{env}. Recognizing that $Q_{env} = Q_{gen,net} - Q_{evap}$, we can also calculate T_s using the two equations in Part IV to see how it matches the initial T_s guess. If the T_S guess matches the calculated T_S values, move on to Step 6. If not, adjust the T_S guess to match the T_S values and return to Step 3. This process is the same as done with the T_{FA} guesses. Thus it is not truly an analytical solution because you need to guess to get to a balance. This guessing is needed since h_c and h_r use T_{FA} and you can't extract T_{FA} from those equations.

Step 6. At this point, the T_{FA} guess used to calculate h_c and h_r in the T_{FA} equation and the T_S guess used to calculate Q_{evap} both match the calculated T_{FA} and T_S values at steady state. Now we know the $Q_{gen,net}$, Q_{fur}, and Q_{env} for an animal in steady state.

Step 7. Check to make sure that everything is working correctly: 1) Do the two T_S calculations give the same skin temperature? 2) Does $Q_{gen,net} - Q_{evap} = Q_{fur} = Q_{env}$?

Step 8. Calculate Q_{resp} by guessing for total Q_{gen} that will result in $Q_{gen} - Q_{resp}$ = the calculated $Q_{gen,net}$. This guessing is needed since Q_{resp} is dependent on total Q_{gen}, not simply the $Q_{gen,net}$. Start at $Q_{gen,net}$, and then increase as needed.

Step 9 Calculate Q_{gen} by adding Q_{resp} from Step 8 to the calculated $Q_{gen,net}$ from Step 6. This is the metabolic heat generation that allows the animal to maintain its body temperature

while remaining in heat balance with its surroundings. Furthermore, the T_S and T_{FA} values are simultaneous functions of animal physiology and environmental conditions.

VII. Notes

The complete Q_{rad} is

$Q_{rad} = Q_{rad,sky} + Q_{rad,grd} + Q_{rad,bush} + Q_{rad,veg}$. Inserting the mechanism equations

$Q_{rad} = h_{r,sky} \cdot A_{FA} \cdot (T_r - T_{sky}) + h_{r,grd} \cdot A_{FA} \cdot (T_r - T_{grd}) + h_{r,bsh} \cdot A_{FA} \cdot (T_r - T_{bush}) + h_{r,veg} \cdot A_{FA} \cdot (T_r - T_{veg})$

where grd is ground, bsh is bush or other nearby object on the ground whose geometry is defined and veg is vegetation overhead. Combining the relevant area, A, and the radiant heat transfer coefficient, h_r, for each of the four terms we can write

$$Q_{rad} = Q_{r1} \cdot (T_r - T_{sky}) + Q_{r2} \cdot (T_r - T_{grd}) + Q_{r3} \cdot (T_r - T_{bush}) + Q_{r4} \cdot (T_r - T_{veg}) \qquad (25)$$

Substituting $(D_3 + T_{FA})$ for T_r:

$Q_{rad} = Q_{r1} \cdot ((D_3 + T_{FA}) - T_{sky}) + Q_{r2} \cdot ((D_3 + T_{FA}) - T_{grd}) + Q_{r3} \cdot ((D_3 + T_{FA}) - T_{bush}) + Q_{r4} \cdot ((D_3 + T_{FA}) - T_{veg})$

Putting this full Q_{rad} into the T_{FA} equation:

$$T_{FA} = \frac{Q_{r1} \cdot T_{sky} + Q_{r2} \cdot T_{grd} + Q_{r3} \cdot T_{bush} + Q_{r4} \cdot T_{veg} + h_c A_{FA} T_A}{(Q_{r1}+Q_{r2}+Q_{r3}+Q_{r4}) + h_c \cdot A_{FA} + \frac{C_f}{D_1}} + \frac{\frac{C_f (T_c - D_2)}{D_1} + Q_{sol} - D_3 (Q_{r1}+Q_{r2}+Q_{r3}+Q_{r4})}{(Q_{r1}+Q_{r2}+Q_{r3}+Q_{r4}) + h_c \cdot A_{FA} + \frac{C_f}{D_1}}$$

VIII. Ellipsoids

When modeling an ellipsoid body shape the following mechanism equations are changed to account for the difference in geometry:

$$Q_{gen,net} = \frac{T_c - T_s}{\frac{S_G^2}{2k_G V_G} + \frac{\left(\sqrt{3S_G^2}\right)^3}{3k_I V_G} \cdot \left(\frac{b_S - b_G}{b_G b_S}\right)} \qquad (26)$$

$$Q_{fur} = \left[\frac{3 \cdot k_F \cdot V_G \cdot b_L \cdot b_S}{\left(\sqrt{3S_G^2}\right)^3 \cdot (b_L - b_S)}\right] \cdot (T_S - T_{FA}) \qquad (27)$$

Derivation of these equations:

1. From the sphere body derivations (Bird et al, 2002):

$$T_G = T_C - \frac{Q_{gen,net} \cdot R_G^2}{2 \cdot k_G \cdot V_G} \qquad (28)$$

The equivalent in an ellipsoid with a:b=c dimensions (Porter and Kearney, 2009):

$$T_G = T_C - \frac{Q_{gen,net} \cdot S_G^2}{2 \cdot k_G \cdot V_G} \qquad (29)$$

where $S_G^2 = \frac{a^2 b^2 c^2}{a^2 b^2 + a^2 c^2 + b^2 c^2}|_G$.

Thus, when converting from sphere geometry to ellipsoid geometry, R_G in a sphere geometry $= \sqrt{3S_G^2}$ in an ellipsoid geometry.

2. In the sphere geometry for a heat generating sphere with an insulating layer (Bird et al, 2002):

$$T_S = T_C - \frac{Q_{gen,net} \cdot R_G^2}{6 \cdot k_G \cdot V_G} - \frac{Q_{gen,net} \cdot R_G^3}{3 \cdot k_I \cdot V_G} \cdot \left(\frac{R_S - R_G}{R_G R_S}\right) \tag{30}$$

So in an ellipsoid geometry:

$$T_S = T_C - \frac{Q_{gen,net} \cdot \left(\sqrt{3S_G^2}\right)^2}{2 \cdot k_G \cdot V_G} - \frac{Q_{gen,net} \cdot \left(\sqrt{3S_G^2}\right)^3}{3 \cdot k_I \cdot V_G} \cdot \left(\frac{b_S - b_G}{b_S \cdot b_G}\right) \tag{31}$$

3. From the fur derivations:
In the sphere geometry where there is a hollow sphere conducting heat, (Bird et al, 2002):

$$T_S = \frac{Q_{gen,net} \cdot R_G^3}{3 \cdot k_F \cdot V_G} \cdot \left(\frac{R_L - R_S}{R_L \cdot R_S}\right) + T_{FA} \tag{32}$$

So in an ellipsoid geometry:

$$T_S = \frac{Q_{gen,net} \cdot \left(\sqrt{3S_G^2}\right)^3}{3 \cdot k_F \cdot V_G} \cdot \left(\frac{b_L - b_S}{b_L \cdot b_S}\right) + T_{FA} \tag{33}$$

Given the above changes for the object's geometry when modeling an ellipsoid compared to a cylinder, the constants used in the heat balance solution are thus changed. The mathematical basis remains the same, as described in the full cylinder solution. All that changes are shape factors used in the mechanism equations mentioned above to account for the different geometries:

$$C_f = \frac{3 \cdot k_F \cdot V_G \cdot b_L \cdot b_S}{\left(\sqrt{3S_G^2}\right)^3 \cdot (b_L - b_S)} \tag{34}$$

$$D_1 = 1 + \frac{C_f \cdot S_G^2}{2 \cdot k_G \cdot V_G} + \frac{C_f \cdot \left(\sqrt{3S_G^2}\right)^3}{3 \cdot k_I . V_G} \cdot \left(\frac{b_S - b_G}{b_S \cdot b_G}\right) \tag{35}$$

$$D_2 = \frac{Q_{evap} \cdot S_G^2}{2 \cdot k_G \cdot V_G} + \frac{Q_{evap} \cdot \left(\sqrt{3S_G^2}\right)^3}{3 \cdot k_I \cdot V_G} \cdot \left(\frac{b_S - b_G}{b_S \cdot b_G}\right) \tag{36}$$

$$D_3 = \left[\frac{\frac{C_f}{D_1} \cdot (T_c - D_2 - T_{FA}) \cdot \left(\sqrt{3S_G^2}\right)^3}{3 \cdot k_f \cdot V_G}\right] \cdot \left(\frac{b_L - b_r}{b_L \cdot b_r}\right) \tag{37}$$

The heat balance solution, eq. 26 and eq. 30, and the solution procedure remains exactly the same.

IX. Re-analysis of Porter and Kearney (2009).

To test this updated solution against the previous version of NicheMapper, we reran the analysis described in Porter & Kearney (2009), comparing NicheMapper metabolic predictions to metabolic measurements taken by Scholander et al. (1950). In the reanalysis shown below, all animals were

modeled as ellipsoids with semi-major (a) :semi-minor (b) axes ratios of 4.5:1 or 1.5:1 and body density set to 1000 kg/m^3, as done in Porter & Kearney (2009). Animal weights and fur depths were the same as in Porter & Kearney (2009), and core temperatures were set to 37C and a wind speed of 0.001 m/s, as done in Porter & Kearney (2009). Fur thermal conductivities for individual species were taken from Scholander (1950), rather than using a single fur thermal conductivity value of 0.209 W/mC, as was done in the previous analysis. Flesh thermal conductivity was fixed at 1.0 W/mC for all animals, and radiant exchange was assumed to take place 85% of the way out to the fur surface from the skin.

These results show how the Niche Mapper performs as a general model. While there are some under- and overestimates of lower critical temperatures, the animals were modeled as generic ellipsoids assuming a uniform fur thickness across the entire animal to illustrate how the basic heat balance approach taken by Niche Mapper works reasonably well across a wide range of animals. Adjustments to model inputs such as animal shape (i.e., the a:b axis ratio) and flesh thermal conductivity produce closer results to those measured in Scholander (1950). Furthermore, the Scholander results presented in the figures below are a best line fit to scattered data. With a single species being tested, metabolic rates at a given temperature varied by up to two fold. For example, at 15°C, metabolic measurements reported for the raccoon varied from 150% of BMR to 300% BMR (Scholander 1950).

These results also show the improvement of the modified heat balance solutions procedure now used by Niche Mapper. For 10 of the 12 model animals, the updated solution procedure performed as well or better than the previous version of Niche Mapper (Porter & Kearney 2009). This illustrates how models are constantly in a "beta" version and we are continually improving how they operate.

Figure 1 shows metabolic rates for six Arctic animals as measured by Scholander (solid triangles) or extrapolated from Scholander data (hollow triangles) compared to metabolic predictions by the 2009 version of Niche Mapper (solid circles) and the current version of Niche Mapper (solid line).

Figure 2 shows Metabolic rates for six tropical mammal as measured by Scholander (solid triangles) or extrapolated from Scholander data (hollow triangles) compared to metabolic predictions by the 2009 version of NicheMapper (solid circles) and the current version of NicheMapper (solid line).

Table 1. Animal properties used in the analysis.

Animal	Mass, *kg*	a:b ratio	Fur depth, *mm*	Fur conductivity, $\frac{W}{mC}$
White fox	4.65	1.5	49.8	0.044
Northern collared lemming	0.06	1.5	19.30	0.050
Eskimo dog pups	12.00	1.5	40.70	0.054
Polar bear cubs	8.90	4.5	44.30	0.063
Ground squirrel	1.10	1.5	14.40	0.035
Least weasel	0.05	1.5	7.40	0.027
White-nosed coati	4.15	4.5	13.25	0.059
Jungle rat	0.27	1.5	2.60	0.014
Marmoset	0.23	1.5	12.76	0.041
Night monkey	0.82	4.5	17.30	0.042
Crab-eating raccoon	1.16	4.5	7.30	0.034
Two-toed sloth	3.80	4.5	35.00	0.077

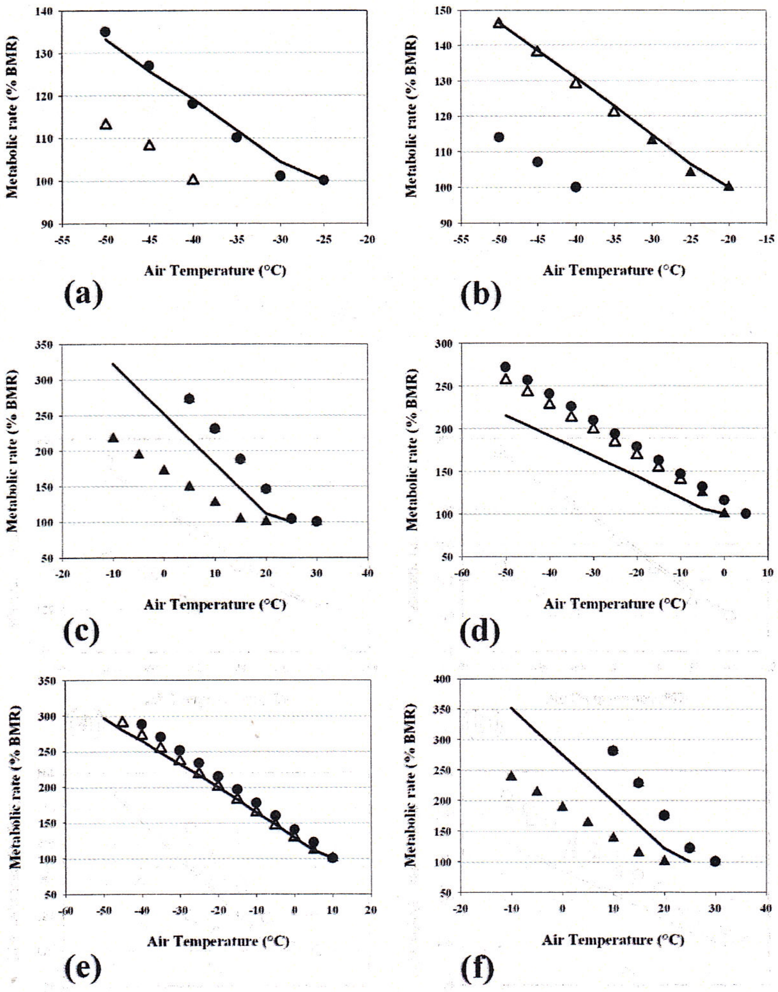

Figure 1: Arctic animals modeled: White fox (a); Eskimo dog pup (b); Lemming (c); Polar bear cubs (d); Ground squirrel (e); and Weasel (f).

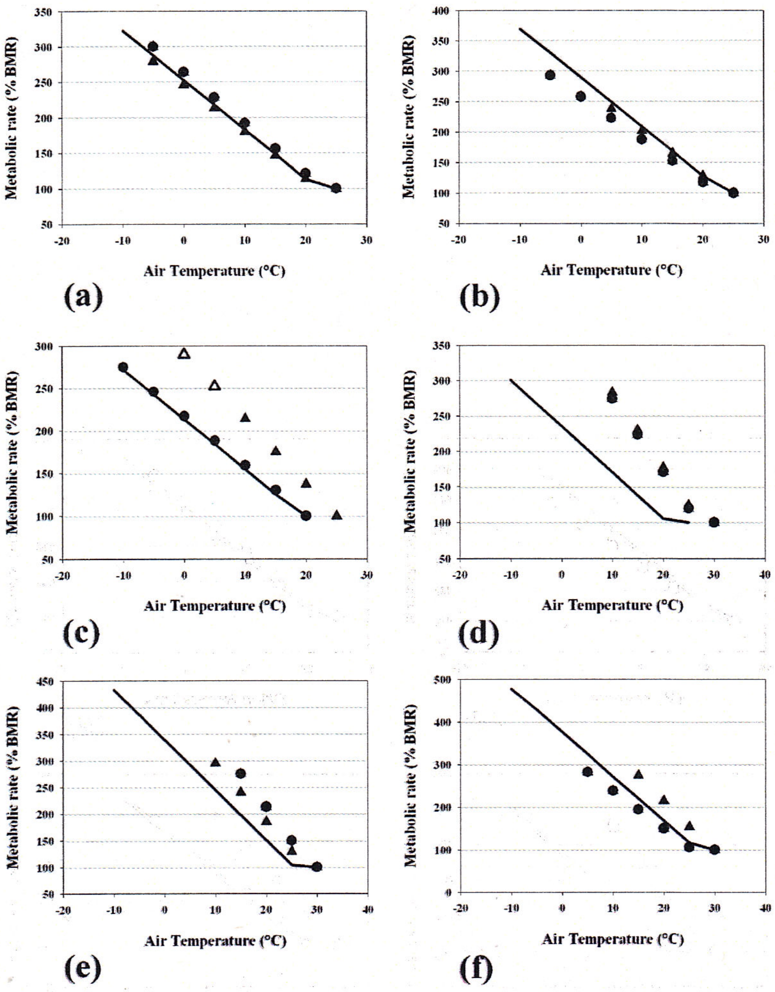

Figure 2: Tropical animals modeled: Coati (a); Jungle rat (b); Marmoset (c); Night monkey (d); Crab-eating raccoon (e); and Two-toed sloth (f).

REFERENCES:

Bird, RB, Stewart, WE, Lightfoot, EN (2002). Transport Phenomena, 2nd ed. Wiley and Sons, New York. 895 pp.

Davis, Jr., LB, Birkebak, RC (1975). Convective energy transfer in fur: 525-548 In: Gates, DM, and Schmerl, RB eds. Perspectives of Biophysical Ecology, Springer-Verlag, New York. 609 pp.

Turnpenny, JR, McArthur, AJ, Clark, JA, Wathes, CM (2000) Thermal balance of livestock: A parsimonious model. Agricultural and Forest Meterology 101: 15-27.

Mount, LE, Brown, D (1982) The use of meteorological records in estimating the effects of weather on sensible heat loss from sheep. Argric. Meteorol. 27: 241-255.

Porter, W.P. and Kearney, M.R.. 2009. Size, shape and the thermal niche of endotherms. PNAS 106 (Suppl. 2) 19666–19672.

Scholander PF, Hock R, Walters V, Johnson F, Irving L (1950) Heat regulation in some arctic and tropical mammals and birds. Biol Bull 99:237–258.

Stafford Smith, DM, Noble, IR, Jones, GK (1985) A heat balance model for sheep and its use to predict shade-seeking behaviour in hot conditions. J. Appl. Ecol. 22: 753-774.

Porter, W.P., Munger, J.C., Stewart, W.E., Budaraju, S. & Jaeger, J. (1994) Endotherm energetics: from a scalable individual-based model to ecological applications. Australian Journal of Zoology, 42, 125-162.

Porter, W.P., Budaraju, S., Stewart, W.E. & Ramankutty, N. (2000) Calculating climate effects on birds and mammals: impacts on biodiversity, conservation, population parameters, and global community structure. Am. Zool., 40, 597-630.

Porter, W.P., Sabo, J.L., Tracy, C.R., Reichman, O.J. & Ramankutty, N. (2002) Physiology on a landscape scale: plant-animal interactions. Integrative and Comparative Biology, 42, 431-453.

Porter, W.P., Vakharia, N., Klousie, W.D. & Duffy, D. (2006) Po'ouli landscape bioinformatics model predicts energetics, behavior, diets, and distribution on Maui. Integrative and Comparative Biology, 46, 1143-1158.
